# A Retinex-based network for image enhancement in low-light environments

**DOI:** 10.1371/journal.pone.0303696

**Published:** 2024-05-24

**Authors:** Ji Wu, Bing Ding, Beining Zhang, Jie Ding

**Affiliations:** 1 School of Electrical and Power Engineering, Taiyuan University of Technology, Taiyuan, China; 2 School of Integrated Circuits and Electronics, Beijing Institute of Technology, Beijing, China; Wuhan University of Science and Technology, CHINA

## Abstract

Most of the existing low-light image enhancement methods suffer from the problems of detail loss, color distortion and excessive noise. To address the above-mentioned issues, this paper proposes a neural network-based low-light image enhancement network. The network is divided into three parts: decomposition network, reflection component denoising network, and illumination component enhancement network. In the decomposition network, the input image is decomposed into a reflection image and an illumination image. In the reflection component denoising network, the Unet3+ network improved by fusion CA attention is adopted to denoise the reflection image. In the illumination component enhancement network, the adaptive mapping curve is adopted to enhance the illumination image iteratively. Finally, the processed illumination and reflection images are fused based on Retinex theory to obtain the final enhanced image. The experimental results show that the proposed network achieves excellent visual effects in subjective evaluation. Additionally, it shows a significant improvement in objective evaluation metrics, including PSNR, SSIM, NIQE, and so on, when compared to the results in several public datasets.

## Introduction

Images play an irreplaceable role in our daily life as a way to obtain information [1]. However, the complicated shooting environment, different lighting conditions and other factors lead to unsatisfactory image acquisition, uneven illumination, low contrast and the presence of a large amount of noise, etc. They interfere with the image recognition in the subsequent processing. The low-light image enhancement technology can be used to make images clearer and reduce identification costs. The research on image enhancement methods in low-light environments is of great significance.

Existing enhancement methods can be classified into two main categories: traditional image enhancement methods and image enhancement methods based on deep learning. Traditional image enhancement methods are mainly used in industry. The main representative methods include grey scale transformation, histogram equalization, and the Retinex method [2–7]. Among them, enhancement methods based on Retinex theory are widely used, such as [5–7]. However, traditional methods are less sensitive to noise. They often result in color distortion and unsatisfied denoising.

Image enhancement methods based on deep learning have developed rapidly in recent years. LLNet was the first application of deep learning theories to low-light image enhancement [8]. Afterwards, Retinex-Net used deep learning neural networks to the Retinex theory for image enhancement [9]. However, they assumed “Ground Truth” image existing and therefore ignored the influence of noise on different regions, resulting bad detail restoration. [10–12] etc. avoided the need for “Ground Truth” reflectance and illumination images, but it overlooked detail optimization, such as structure and texture. To reduce reliance on paired datasets, some unsupervised methods were proposed. Zero-DCE became the first low-light enhancement network that operated independent of paired datasets [13]. EnlightenGAN further minimized reliance on paired data [14]. However, due to the lack of guidance from paired datasets, unsupervised methods were unable to effectively learn real-world scene features and had limited generalization capabilities. Other research methods [15–21] attempted to address the issues of unrealistic recovery effects and complex network scales by introducing new learning modules and attention mechanisms. Nevertheless, these methods had limitations in their experimental outcomes and lack robust generalization. For example, in different scenarios, the restoration effects of [13–16, 18, 21] were unstable, and the models lacked constraints and guidance for specific scenes. When strong light conditions occur, over-enhancement phenomena can be observed in [17, 19, 20]. To address these limitations, we propose a new model based on Retinex. Compared to other advanced methods, the method proposed in this paper shows good restoration effects in complicated conditions such as extreme low-light and over-exposure. It also shows excellent performance in image denoising and enhancement without the need of a large amount of training data.

This paper is arranged as follows. The Proposed Network section introduces the structure of proposed enhanced network. Experimental data sets and details of training sets are presented in the Experimental Process section. The Results and Analysis section analyses the experimental results of different methods. The Conclusions section draws the conclusion.

### Proposed network

**Fig 1:**
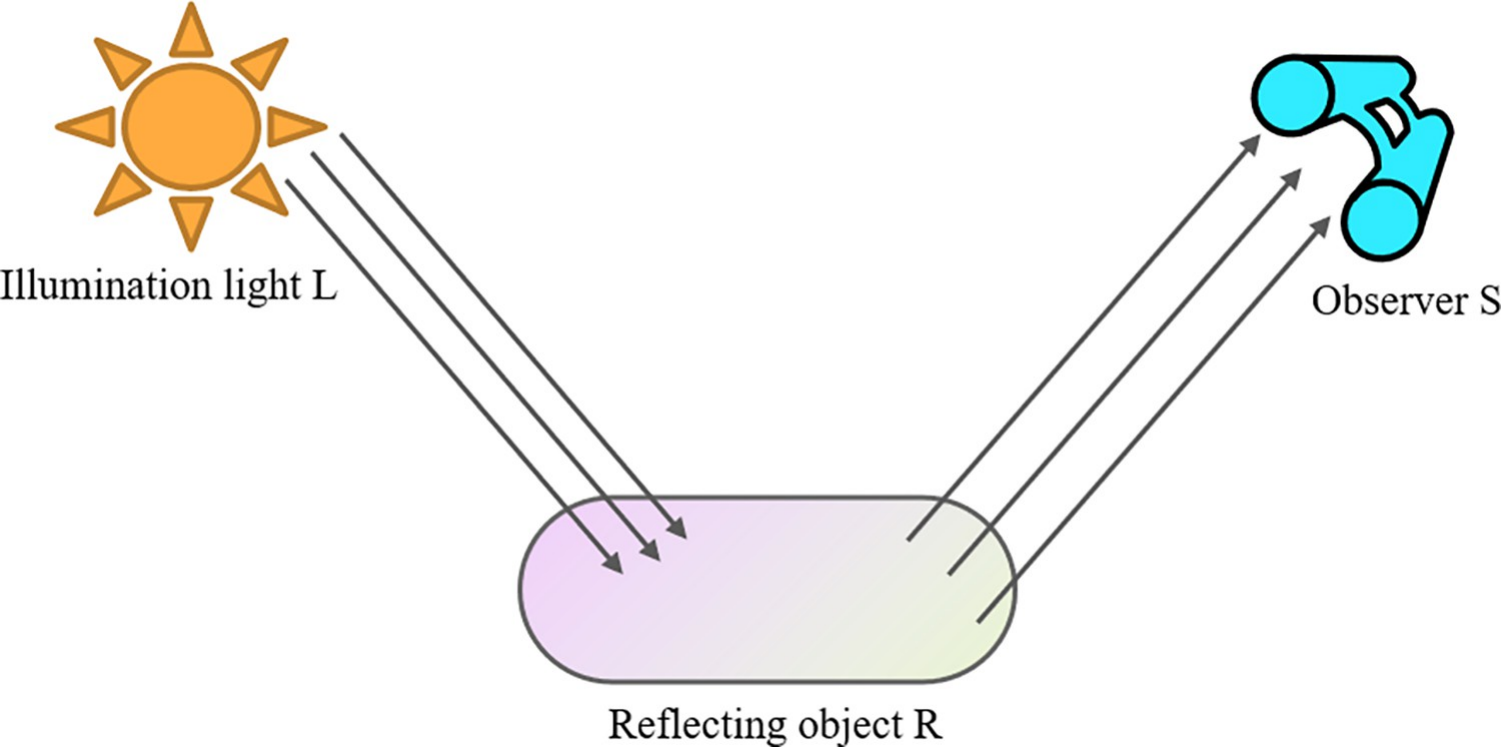
Retinex schematic diagram.

In this paper, a low-light image enhancement network combining Retinex theory [4] and a convolutional neural network is designed. The principle of Retinex is shown in Fig 1.

According to the Retinex theory, the illumination image represents the lighting conditions and the reflection image represents the texture information of the object. The enhancement of the original image is achieved by multiplying the illumination image and the reflection image. This relationship is expressed by Eq (1).


S(x,y)=R(x,y)⋅L(x,y) (1)

*S*(*x*,*y*) represents the image information received by the observer S. *L*(*x*,*y*) represents the illumination component of light. *R*(*x*,*y*) represents the reflection component of the object R.

**Fig 2:**
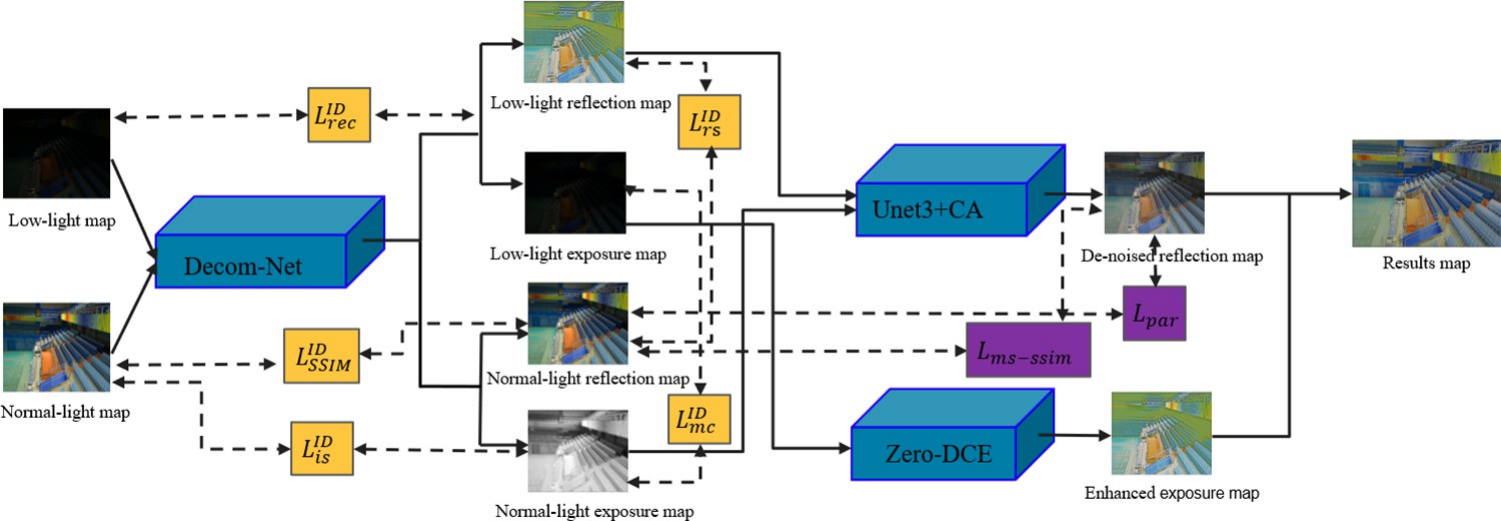
Overall network structure.

Based on the Retinex theory, an image can be decomposed into a reflection component and an illumination component. For each component, a network is built. And an additional network is also needed to decompose the image. Therefore, the proposed network can be divided into three parts: the decomposition network, the reflection component denoising network, and the illumination component enhancement network. The overall network structure is shown in Fig 2. The specific network design and the corresponding loss function for each sub-network are demonstrated in the following part.

### Decomposition network

The structure of the KinD [10] is adopted in the decomposition network. However, the KinD has problems of over-enhancement and visual defects. Therefore, in the first and third convolutional layers, the original activation function ReLU is replaced by the GELU, which exhibits stable optimization capabilities and excellent generalization. Compared to ReLU function, GELU function can better capture complex relationships in image data, aiding in enhancing the structural and textural information of the image. The smoothness of the GELU function reduces issue like gradient explosion or disappearance, resulting in superior performance in preserving image detail information and handling exposure.

**Fig 3:**
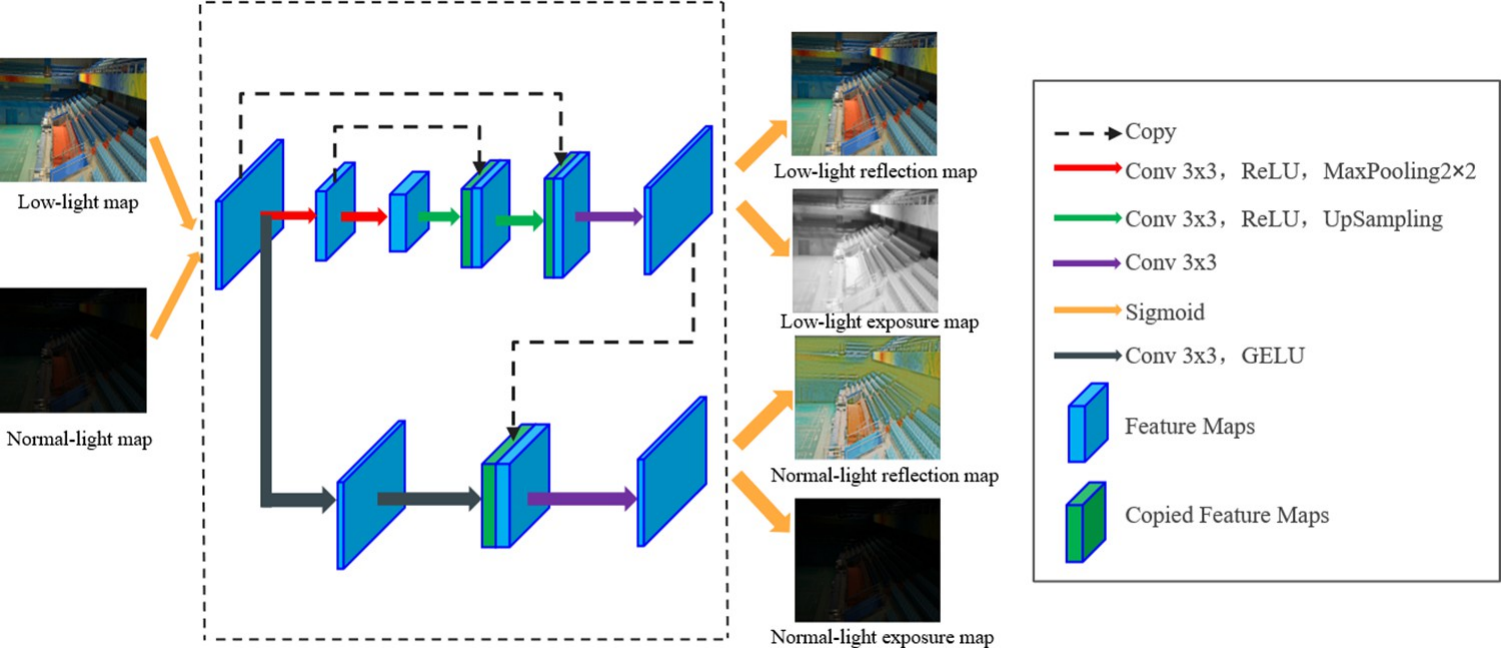
Structure of the decomposition network.

Furthermore, to improve the accuracy of the network, a new structural similarity loss function SSIM [22] is added. SSIM is a metric used to measure image quality. It is mainly used to assess the structural similarity between two images. The SSIM loss function includes three aspects of image features: brightness, contrast and structure. By minimizing the SSIM loss function, the decomposed image can be made closer to the original image and can maintain a better perceptual quality. The details of the decomposition network are shown in Fig 3.

5 loss functions are used in the decomposition network. They are reconstruction loss function, reflection component consistent loss function, illumination component smoothing loss function, illumination intercorrelation loss function, and structural similarity loss function. The details of these loss functions are illustrated below.

The reconstruction loss $L_{rec}^{ID}$ is

$$L_{rec}^{ID}={\left\|S_{l}-R_{l}\cdot I_{l}\right\|}_{1}+{\left\|S_{h}-R_{h}\cdot I_{h}\right\|}_{1}$$ (2)

*S*_*l*_ and *S*_*h*_ denote the low-light image and the normal-light image, respectively. *R*_*l*_ and *R*_*h*_ denote the reflection component from the decomposition of the low-light image and the normal-light image, respectively. *I*_*l*_ and *I*_*h*_ denote the illumination component from the decomposition of the low-light image and the normal-light image, respectively.

The reflection component consistent loss $L_{rs}^{ID}$ is

$$L_{rs}^{ID}={\left\|R_{l}-R_{h}\right\|}_{1}$$ (3)

The illumination component smoothing loss $L_{is}^{ID}$ is

$$L_{is}^{ID}={\left\|\frac{\nabla I_{l}}{max(|\nabla S_{l}|,\epsilon )}\right\|}_{1}+{\left\|\frac{\nabla I_{h}}{max(|\nabla S_{h}|,\epsilon )}\right\|}_{1}$$ (4)

∇ is the first-order derivative operator. *ϵ* is a constant, here it is set to 0.01.

The illumination intercorrelation loss $L_{mc}^{ID}$ is

$$L_{mc}^{ID}={\left\|G\cdot exp(-c\cdot G)\right\|}_{1}$$ (5)

*c* is the parameter that controls the shape of the function, here it is set to 10. G represents the sum of the gradients.

The structural similarity loss $L_{SSIM}^{ID}$ is

$$L_{SSIM}^{ID}=1-\frac{1}{N}{\left\|SSIM(S_{out},S_{h})\right\|}^{2}$$ (6)

$$SSIM(S_{out},S_{h})=\frac{\left(2\mu_{S_{out}}\mu_{S_{h}}+c_{1})(2\sigma_{S_{out}S_{h}}+c_{2}\right)}{\left(\mu_{S_{out}}^{2}+\mu_{S_{h}}^{2}+c_{1})(\sigma_{S_{out}}^{2}+\sigma_{S_{h}}^{2}+c_{2}\right)}$$ (7)

*S*_*out*_ and *S*_*h*_ denote the output image and the normal-light image, respectively. $\mu_{S_{out}}$ and $\mu_{S_{h}}$ denote the mean values of the output image and the normal-light image, respectively. $\sigma_{S_{out}}$ and $\sigma_{S_{h}}$ denote the standard deviation of the output image and the normal-light image, respectively. *c*_1_ and *c*_2_ are constants.

The total loss function of the image decomposition network is

$$L^{ID}=\lambda_{rec}L_{rec}^{ID}+\lambda_{rs}L_{rs}^{ID}+\lambda_{is}L_{is}^{ID}+\lambda_{mc}L_{mc}^{ID}+\lambda_{SSIM}L_{SSIM}^{ID}$$ (8)

λ_rec_, λ_rs_, λ_is_, λ_mc_, and λ_SSIM_ are the weighting coefficients for reconstruction loss, reflection component agreement loss, illumination component smoothing loss, illumination intercorrelation loss, and structural similarity loss, respectively. λ_rec_, λ_rs_, λ_is_, λ_mc_, and λ_SSIM_ are set to 1, 0.009, 0.2, 0.15 and 0.07, respectively.

**Fig 4:**
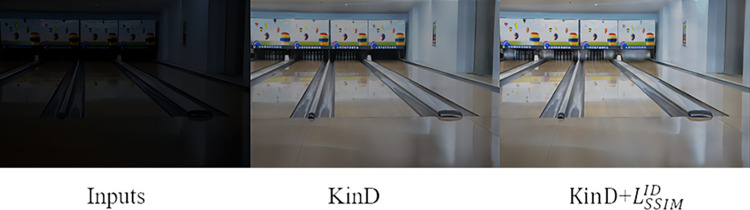
Results of adding SSIM loss function to KinD.

We compare the effects before and after adding the SSIM loss function to KinD. The experimental results are shown in Fig 4. Although KinD has a muddy shadow in some areas after adding SSIM loss function, the overall effect is better. The outcome aligns more closely with the visual perception of the human eye.

**Fig 5:**
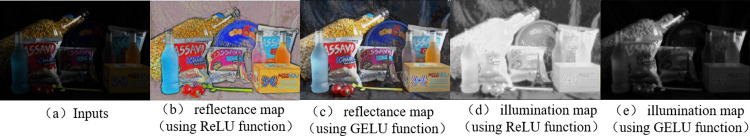
Comparison of ReLU function and GELU function in decomposition network.

Fig 5 shows the effects of replacing the ReLU function with the GELU function in the decomposition network. Fig 5(B) depicts that the reflection image is blurry and has serious color distortion phenomenon when using ReLU. Contrarily, in Fig 5(C), using GELU results in more realistic colors and less noise. Fig 5(D) shows overexposure in the illumination image when using ReLU, whereas Fig 5(E) depicts the image clearer when using GELU.

### Reflective component denoising network

**Fig 6:**
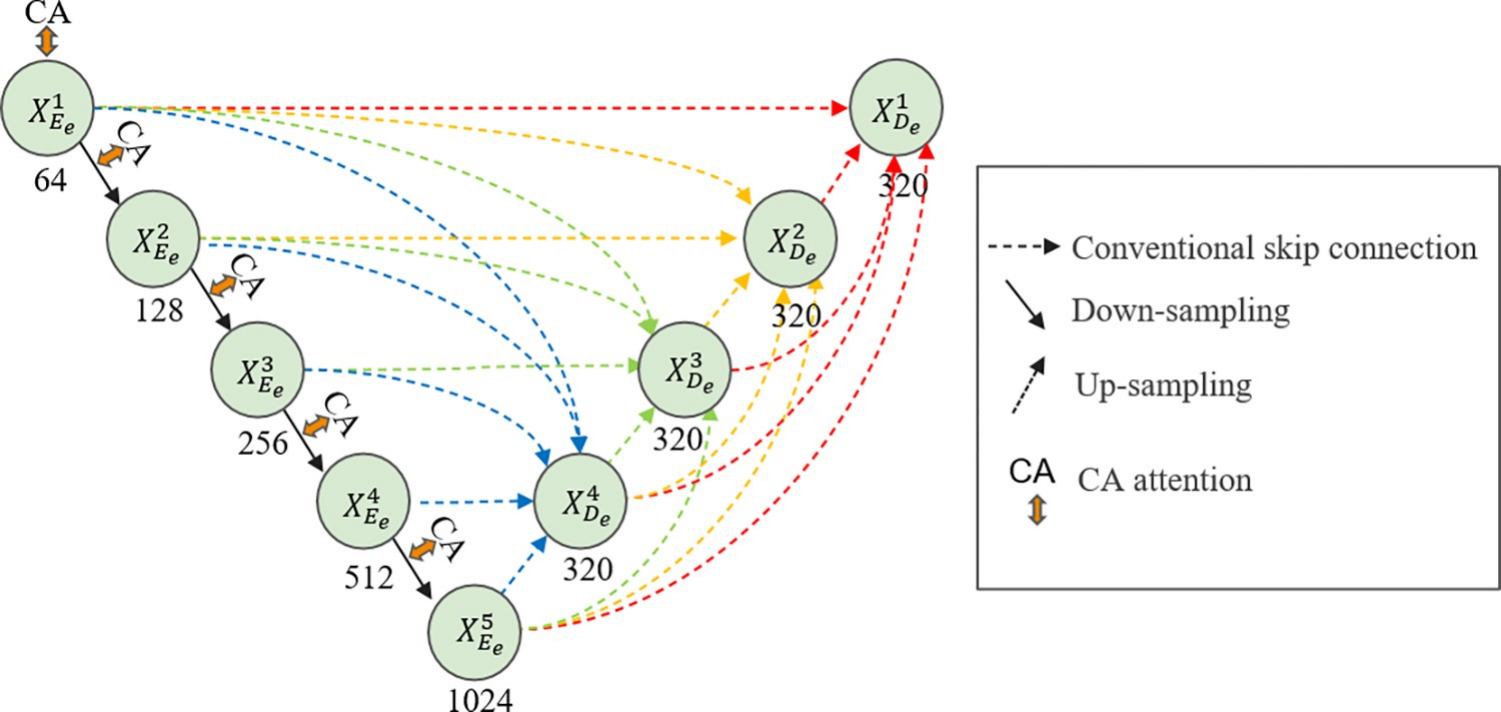
Structure of the reflective component denoising network.

When the low-light image passes through the decomposition network, the reflection image retains the detail information. However, the noise in the low-light region is amplified at the same time. Therefore, it is necessary to denoise the decomposed reflection image. The structure of the Unet3+ [23] is adopted in the reflective component denoising network. However, the Unet3+ does not consider the extracting object size, which results in a mismatch between the receptive field and the scale. It leads to certain limitations in denoising. Therefore, CA attention [24] is added to the encoder part in Unet3+. CA attention combines channel attention and spatial attention to enhance the capture of direction and position information. It can help the network to adaptively learn the noise model of different regions in the image and make weighted estimates of the noise so that the network can more accurately recover parts of the signal and retain more detailed information. CA Attention can help the network to achieve local attention, allowing the network to focus more on the regions in the image that need to be processed. Meanwhile, it can be easily inserted into the network module to improve accuracy. The details of the reflective component denoising network are shown in Fig 6.

2 loss functions are used in the reflective component denoising network. They are multi-scale structural similarity loss function and detail loss function. The details of these loss functions are illustrated below.

The multi-scale structural similarity loss *L*_*ms−ssim*_ is

$$L_{ms-ssim}=1-\prod_{m=1}^{M}\begin{array}{c} {\left(\frac{2\mu_{p}\mu_{g}+C_{1}}{\mu_{p}^{2}+\mu_{g}^{2}+C_{1}}\right)}^{\beta_{m}}\\ {\left(\frac{2\sigma_{pg}+C_{2}}{\sigma_{p}^{2}+\sigma_{g}^{2}+C_{2}}\right)}^{\gamma_{m}} \end{array}$$ (9)

*M* denotes the total number of scales, here it is set to 2. *μ*_*p*_ and *μ*_*g*_ denote the mean of the denoised and normal-light reflectance images, respectively. *σ*_*p*_ and *σ*_*g*_ denote the standard deviation of the denoised and normal-light reflectance images, respectively. *C*_1_ and *C*_2_ are constants. *σ*_*pg*_ denotes the covariance of the denoised and normal-light reflectance images. Both the *β*_*m*_ and *γ*_*m*_ components are set to 0.2856.

The detail loss *L*_*par*_ is

$$L_{par}={\left\|R_{h}-R_{L}\right\|}_{1}$$ (10)

*R*_*h*_ denotes the reflectance image of the normal-light image. *R*_*L*_ denotes the denoised reflectance image. || ||_1_ denotes the L_1_ parametric regularization constraint on both.

The total loss function of the reflectance component denoising network is

$$L_{seg}={\lambda_{ms-ssim}L}_{ms-ssim}+\lambda_{par}L_{par}$$ (11)

λ_*ms−ssim*_ and λ_par_ are the weighting coefficients of the multi-scale structural similarity loss and detail loss, respectively. λ_*ms−ssim*_ is set to 1 and λ_par_ is set to 0.009.

**Fig 7:**
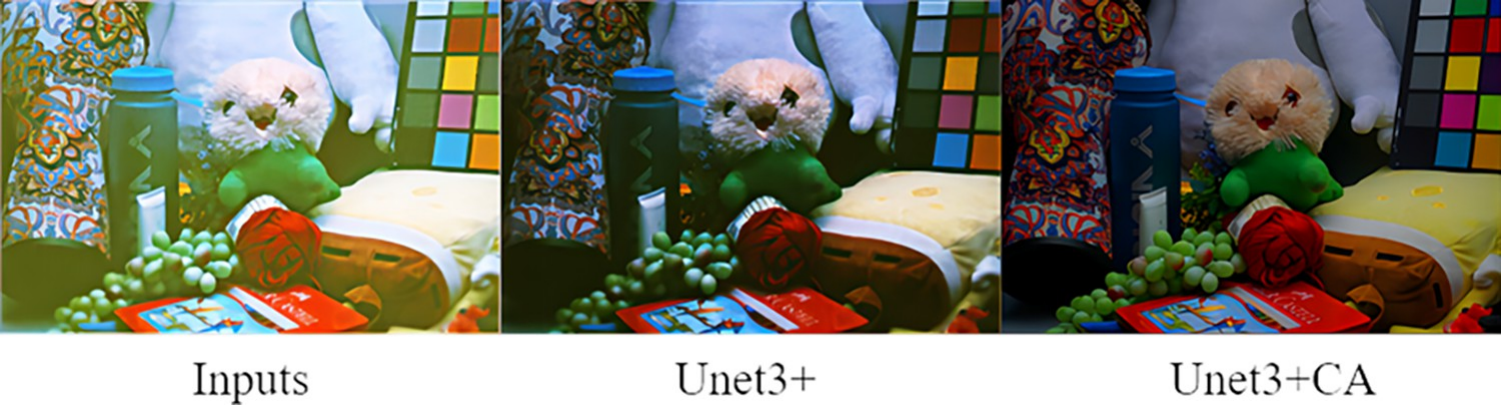
Results of adding CA attention to Unet3+.

We compare the effects before and after adding the CA attention to Unet3+. The experimental results are shown in Fig 7. The Unet3+ recovered image is brighter, but it suffers from increased blurriness and noise. Although the addition of CA attention reduces image brightness, it significantly improves denoising effect. The colors in image are fuller and more realistic.

### Illumination component enhancement network

**Fig 8:**
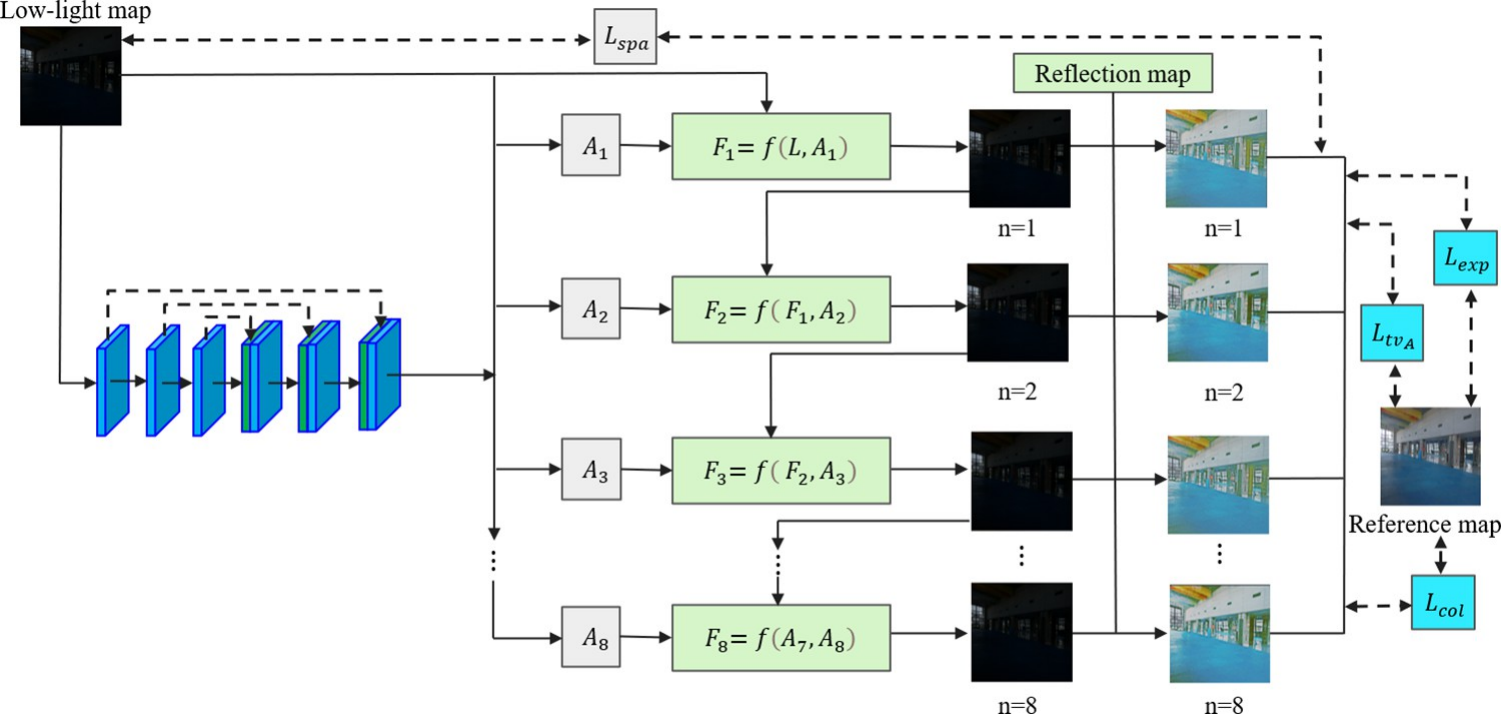
Structure of the illumination component enhancement network.

The illumination image represents different light distributions in the image. Zero-DCE has the advantages of lightweight and excellent image brightness enhancement. Though Zero-DCE’s denoising effect is ordinary, the reflection component denoising network proposed in this paper has a good denoising effect. Therefore, Zero-DCE is adopted in the illumination component enhancement network. Zero-DCE is used to improve the curve fit through multiple iterations. The difference in the experimental results. Therefore, n is set to find the best iteration times through multiple experiments in this work. The details of the illumination component enhancement network are shown in Fig 8.

4 loss functions are used in the illumination component enhancement network. They are exposure control loss function, color constant loss function, illumination smoothing loss function, and spatial consistency loss function. The details of these loss functions are illustrated below.

The exposure control loss *L*_*exp*_ is

$$L_{exp}=\frac{1}{M}\sum_{k=1}^{M}\left|Y_{k}-E\right|$$ (12)

*E* is a constant. It is set to 0.6. *M* is the toal number of pixels. *Y*_*k*_ is the mean value of a pixel region.

The color constant loss *L*_*col*_ is

$$L_{col}=\sum_{\forall (p,q)\in \varepsilon }{\left(J^{p}-J^{q}\right)}^{2},\varepsilon =\{\left(R,G),(R,B),(G,B\right)\}$$ (13)

*J*^*p*^ and *J*^*q*^ are the luminance averages of color channel p and color channel q, respectively. (p,q) traverses all two-by-two combinations of three color channels.

The illumination smoothing loss $L_{{tv}_{A}}$ is

$$L_{{tv}_{A}}=\frac{1}{N}\sum_{n=1}^{N}\sum_{c\in \xi }{\left(\left|\nabla_{x}A_{n}^{c}|+|\nabla_{y}A_{n}^{c}\right|\right)}^{2},\xi =\{R,G,B\}$$ (14)

*N* denotes the iteration times. ∇_*x*_ and ∇_*y*_ denote the gradient operators in the horizontal and vertical directions, respectively.

The spatial consistency loss *L*_*spa*_ is

$$L_{spa}=\frac{1}{M}\sum_{i=1}^{M}\sum_{j\in \Omega (i)}{\left(\left|\left(Y_{i}-Y_{j}\right)|-|\left(I_{i}-I_{j}\right)\right|\right)}^{2}$$ (15)

*Y* denotes the pixel value after enhancement. *I* denotes the pixel value before enhancement. *Ω* is the neighboring pixels of the pixel.

The total loss function of the illumination component enhancement network is

$$L_{total}=W_{exp}L_{exp}+{W_{col}L}_{col}+W_{{tv}_{A}}L_{{tv}_{A}}+W_{spa}L_{spa}$$ (16)

$W_{exp},W_{col},W_{{tv}_{A}}$, and W_*spa*_ are the weighting factors for exposure control loss, color constancy loss, illumination smoothing loss, and spatial consistency loss, respectively. $W_{exp},W_{col},W_{{tv}_{A}}$, and W_*spa*_ are set to 10, 5, 200 and 1, respectively.

**Fig 9:**
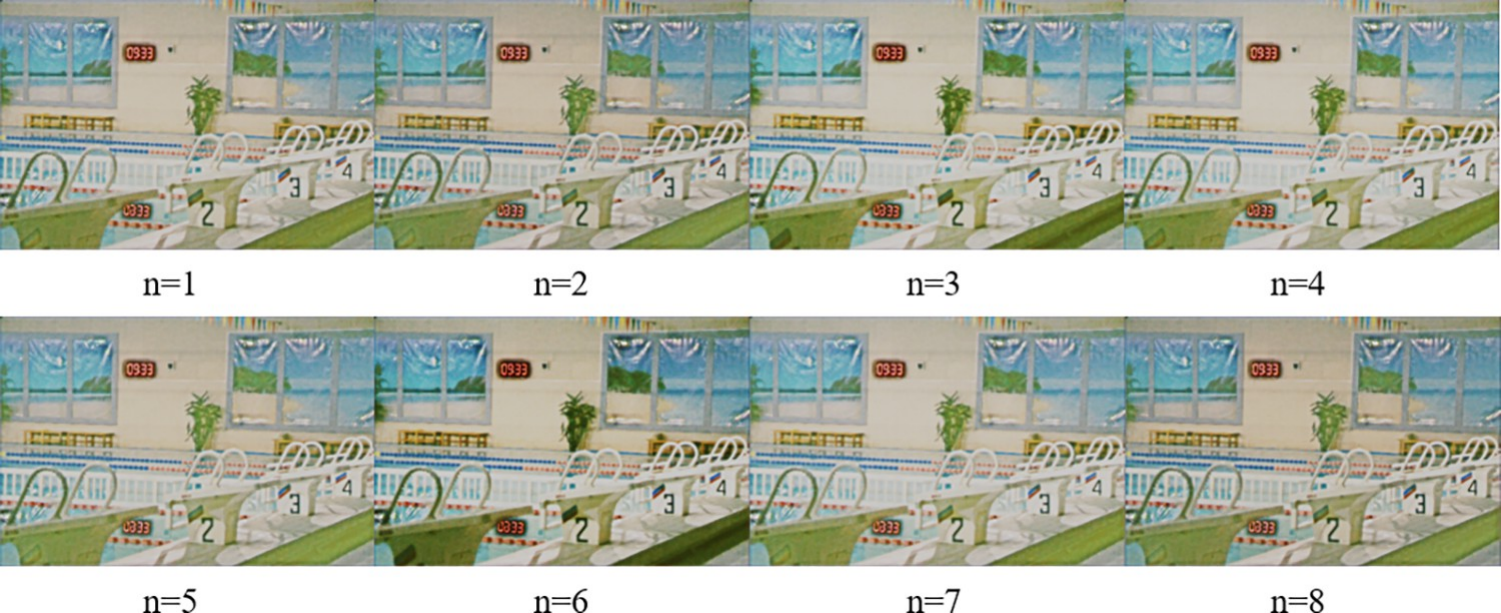
Comparison results of different iteration times n.

The results of different iteration times in the illumination component enhancement network are shown in Table 1 and Fig 9. Table 1 shows that the effect reaches the best when n is 6. Fig 9 also verifies that when n is 6 the image is more in line with the human subjective visual effect.

**Table 1:** Comparison results of different iteration times n.

Iteration times	PSNR	SSIM	NIQE	PI	BRISQUE	NIMA
**n = 1**	15.3598	0.6418	5.4707	4.9396	25.583	3.9284
**n = 2**	15.6933	0.6453	5.3707	4.8745	19.6636	3.935
**n = 3**	16.7013	0.6447	5.535	4.9694	29.8842	3.9314
**n = 4**	18.9715	0.65	5.3844	4.9001	20.7595	3.9296
**n = 5**	19.0601	0.6484	5.262	4.7865	17.5148	3.9537
**n = 6**	**20.4757**	**0.6962**	**5.2264**	4.7918	20.4704	**3.9665**
**n = 7**	19.4365	0.6528	5.4927	4.9228	29.5423	3.9387
**n = 8**	19.233	0.6484	5.2708	**4.7793**	**17.1984**	3.9621

## Experimental process

### Experimental data sets

In the training process, 485 groups of LOL dataset are used as the training set. The remaining 15 groups of LOL dataset are used as the test set. In order to verify the model effect, the paired datasets VE-LOL-L, SID and ELD are used as other test sets. The unpaired datasets DICM and MEF are also used as the test set.

### Details of training process

The experiments are carried out under the framework Pytorch 1.10.1, based on Python 3.7 with Cuda 11.1 environment. The Adam optimizer is used in the training process. The low-light image enhancement is accomplished by the proposed three sub-networks. The experimental details and steps are illustrated below.

1. Input the normal-light image and the low-light image into the decomposition network for decomposition. The learning rate of the decomposition network is set to 0.004. The batch size is set to 32.
2. Input the decomposed reflection image into the reflection component denoising network for denoising. The learning rate of the reflection component denoising network is set to 0.001. The batch size is set to 1.
3. Input the decomposed illumination image into the illumination component enhancement network for enhancement. The learning rate of the illumination component enhancement network is set to 0.001. The iteration times n is set to 6. The batch size is set to 8.
4. Multiply the denoised reflection image R(x,y) and the enhanced image L(x,y) to obtain the final image.

## Results and analysis

Both subjective and objective visual evaluations are employed to evaluate the effects of image enhancement. To validate the necessity of each sub-network, we have conducted ablation experiments. In the objective visual evaluation, various representative metrics are used to assess the experiments. These metrics include peak signal-to-noise ratio (PSNR), structural similarity (SSIM), and no- reference metrics, such as Natural Image Quality Evaluation (NIQE), Image Perceptual Quality (PI), No- Reference Quality Evaluation (BRISQUE), and Neural Image Assessment (NIMA).

### Subjective visual evaluation

**Fig 10:**
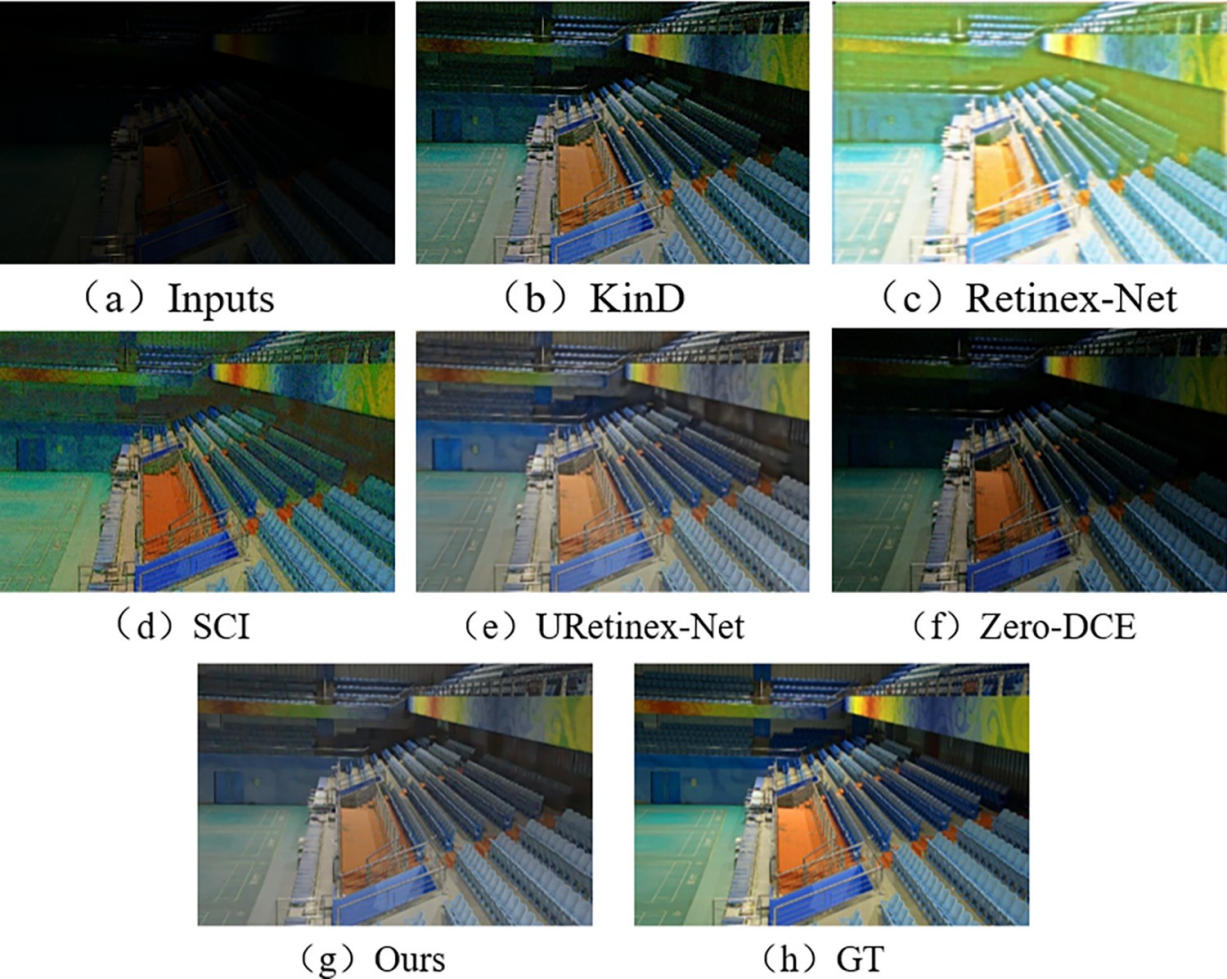
Comparison results of different methods on LOL test set.

**Fig 11:**
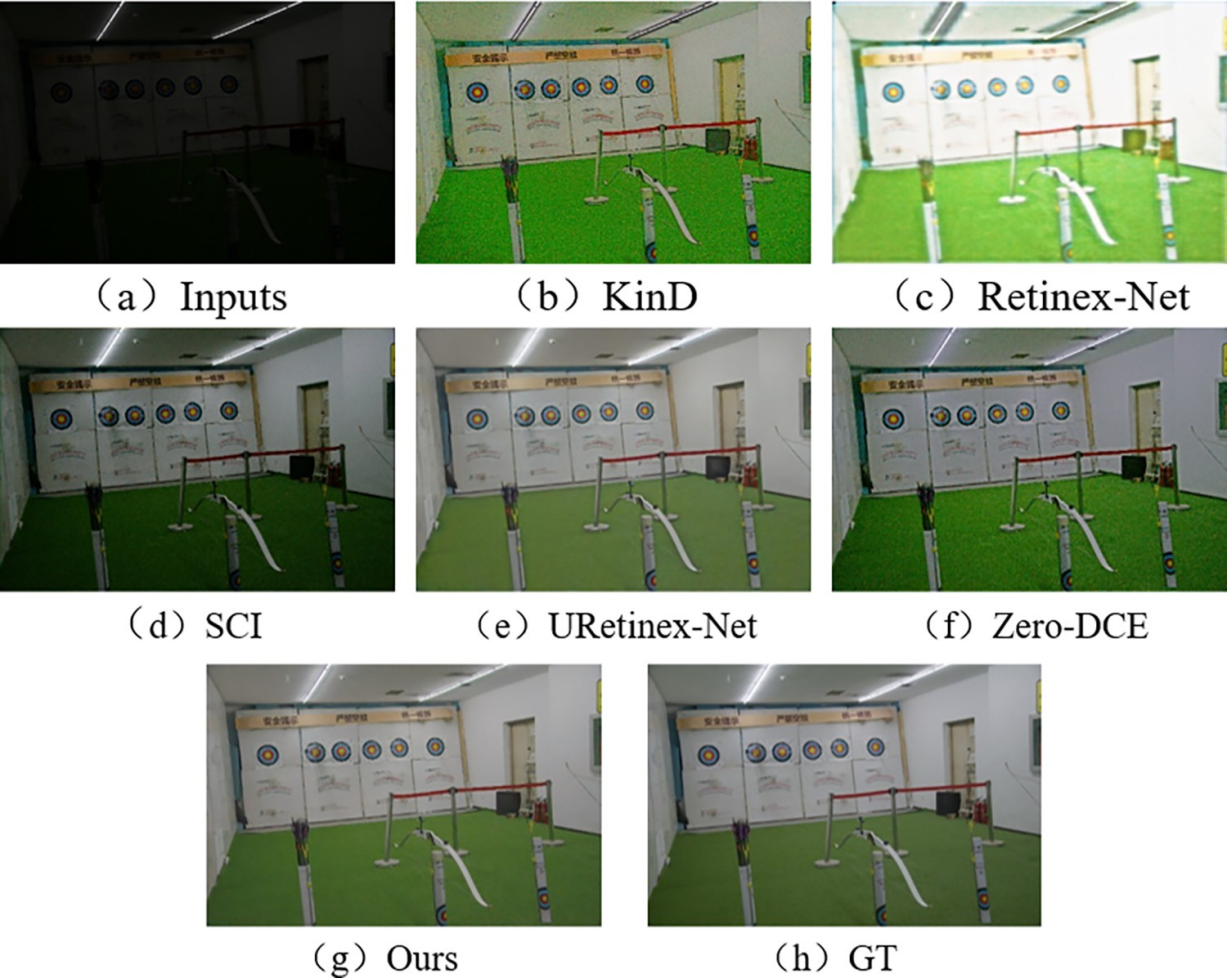
Comparison results of different methods on VE-LOL-L test set.

**Fig 12:**
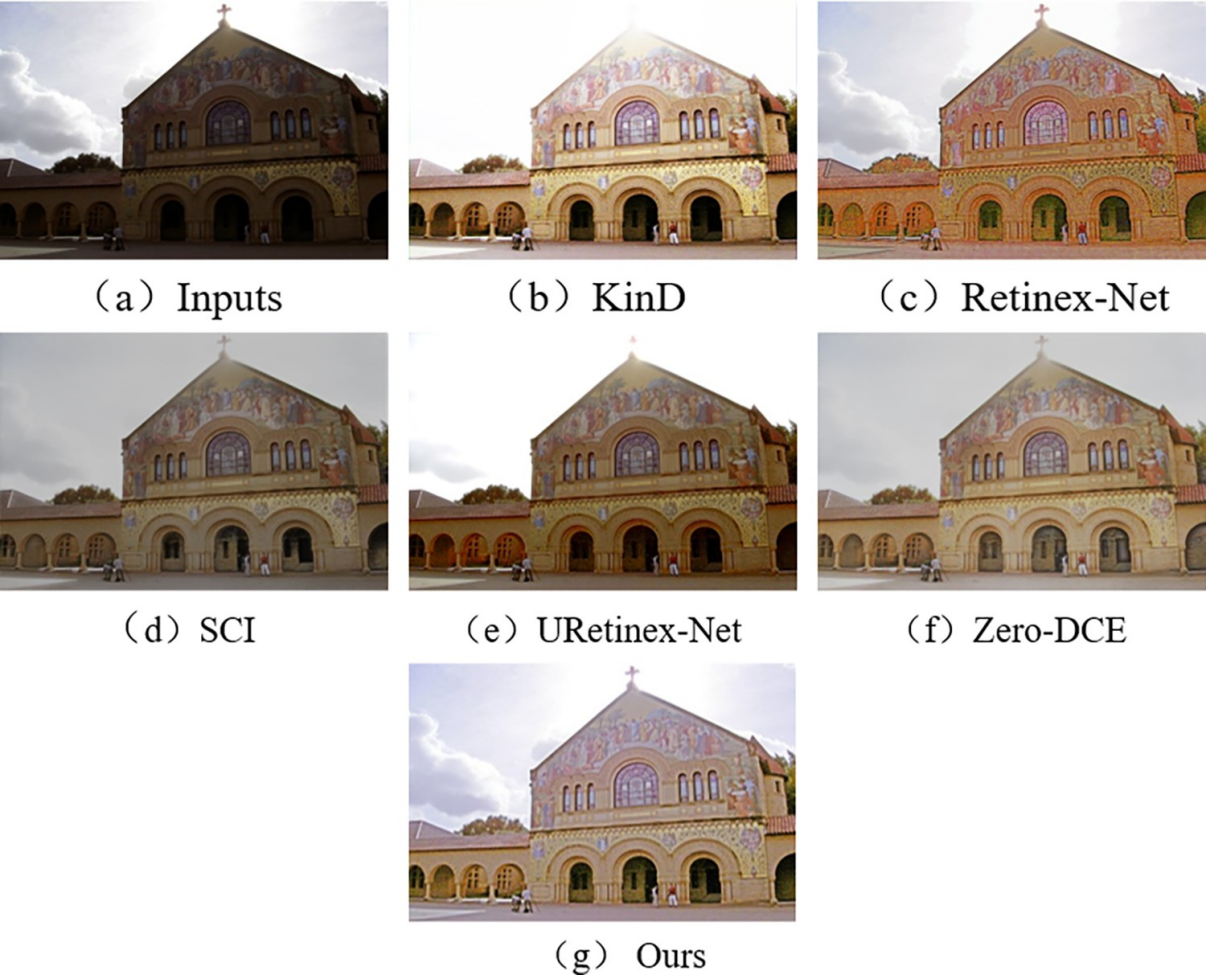
Comparison results of different methods on DICM test set.

**Fig 13:**
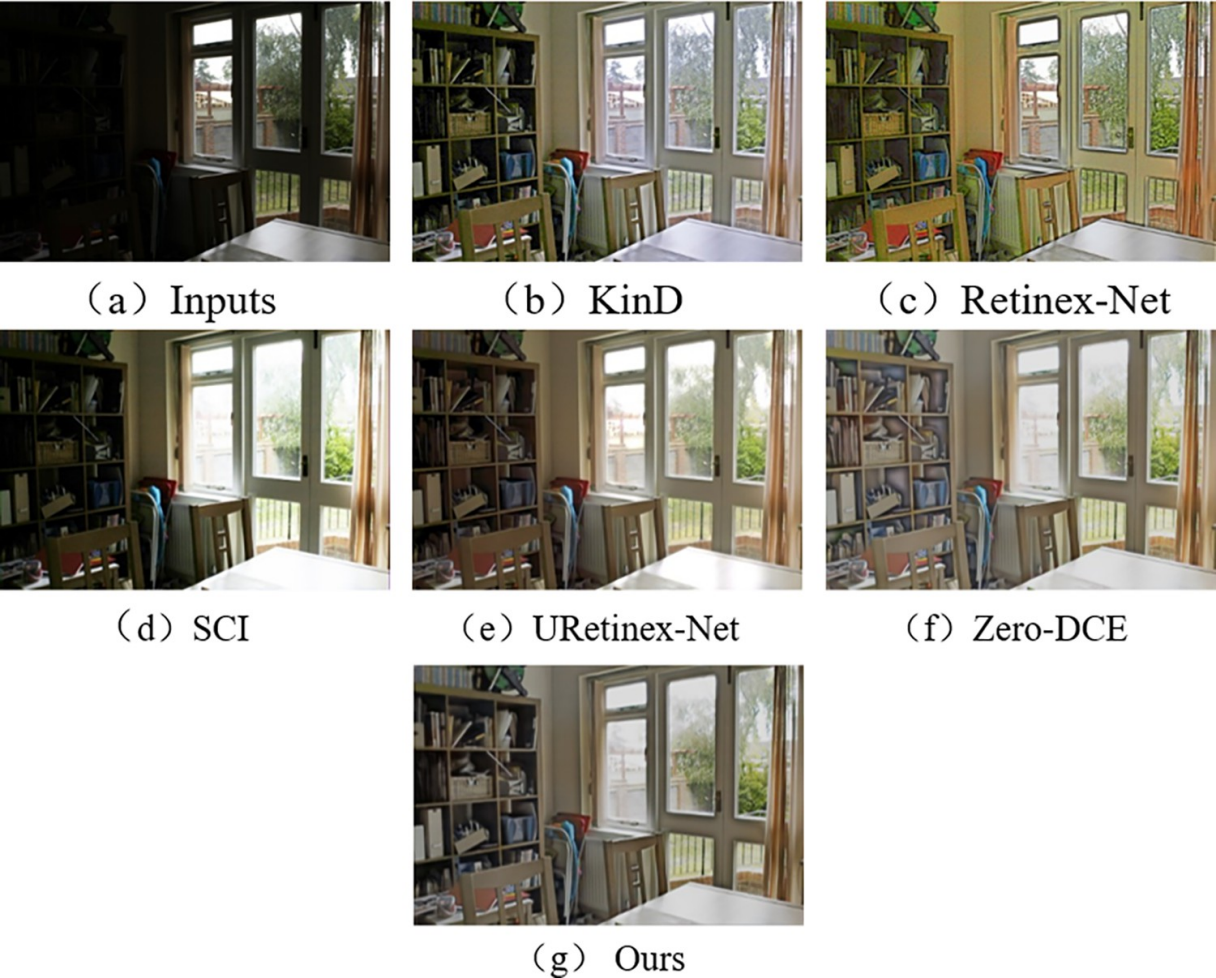
Comparison results of different methods on MEF test set.

**Fig 14:**
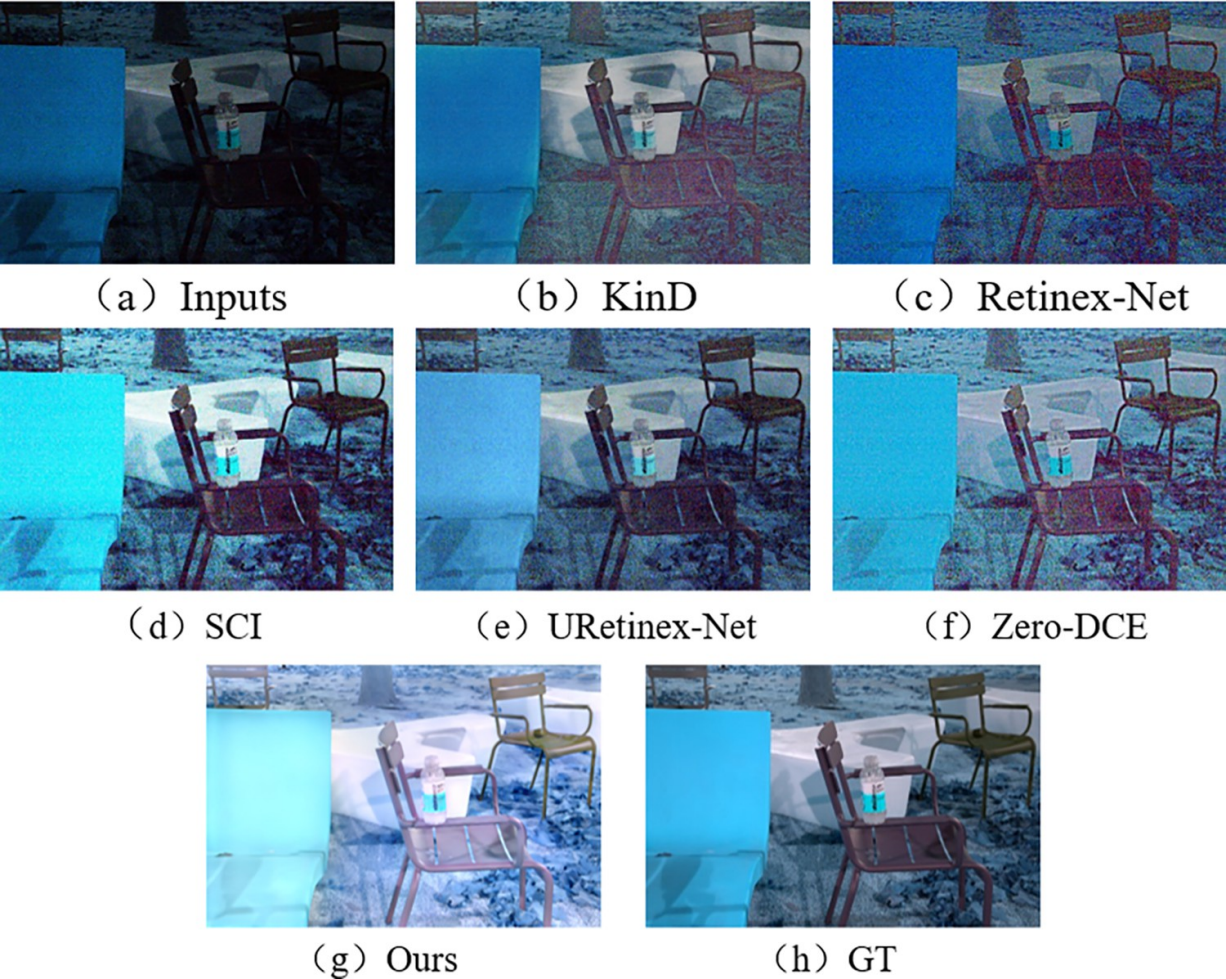
Comparison results of different methods on SID test set.

**Fig 15:**
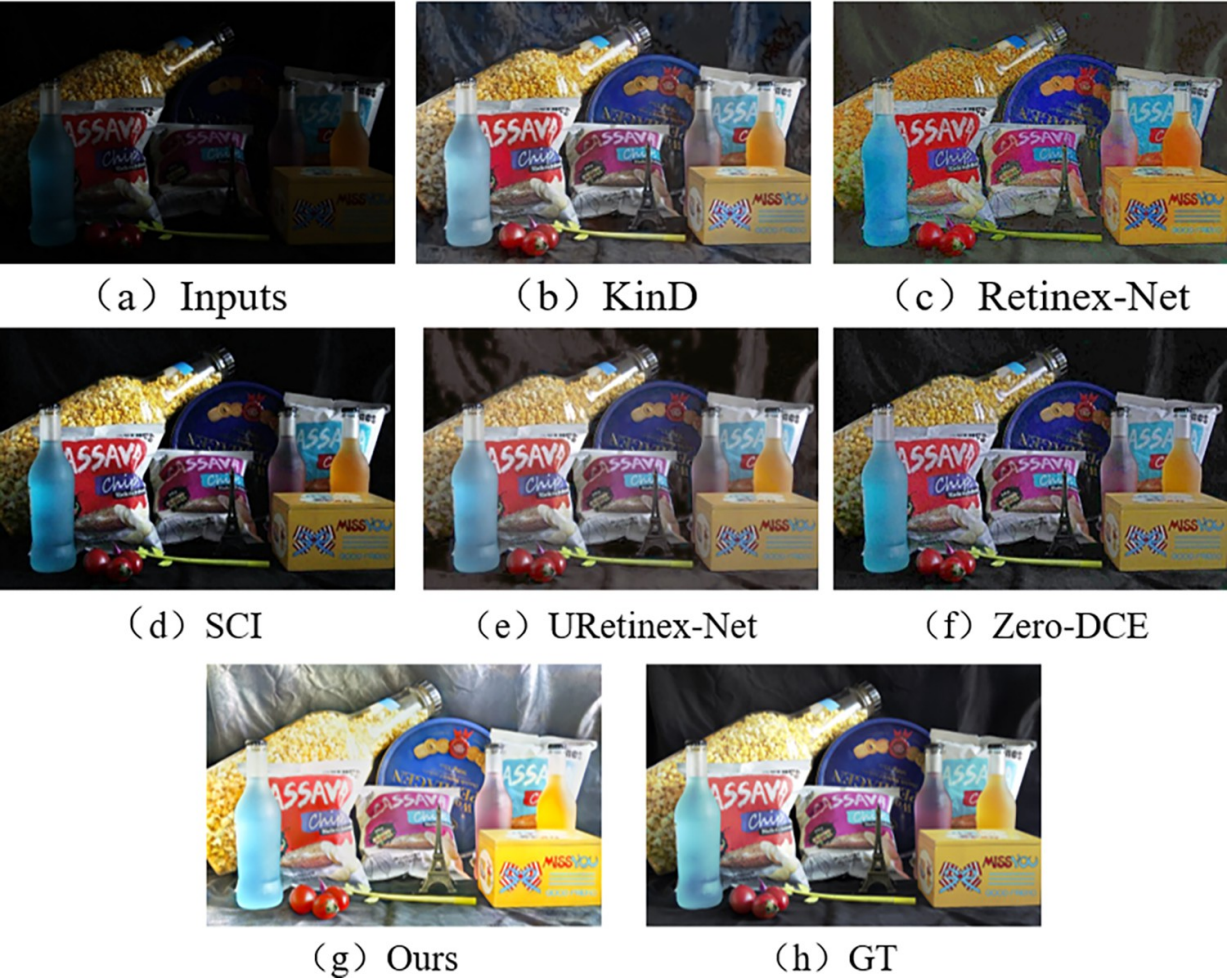
Comparison results of different methods on ELD test set.

In the subjective visual evaluation, KinD, Retinex-Net, SCI, URetinex-Net, and Zero-DCE are selected for comparison. The final results on the test set are shown in Figs 10–15.

From Figs 10–15, it can be seen that the proposed network and rest of five methods in the comparison are all able to achieve low-light images enhancement. However, each method has its own disadvantages. Figs 10(C) and 13(C) show that the Retinex-Net enhanced images are too noisy. There is a serious distortion in the color recovery, and the overall effect is unsatisfied. The Retinex-Net is very likely to lead to serious global color distortion. Although some of the noise can be removed, the global color shift is obvious.

Figs 10(B) and 12(B) show overexposure when recovering a brighter light source using KinD. As shown in Fig 15(B), the upper part of the image is also over-enhanced. The KinD can lead to image over-enhancement or distortion. It is not in line with the actual scene.

As shown in Fig 11(D), the SCI has a dim recovery effect on indoor archery image. Fig 13(D) also uses SCI enhancement method. However, the result of SCI is darker in the indoor bookshelf area when compared with results obtained from other methods. As shown in Fig 15(D), the brightness of the black background area in the image is almost not enhanced at all, but in Fig 14(D), it is over-enhanced. It has a significant difference from the image under normal-light. SCI can lead to abnormal brightness enhancement in some cases. It makes the image unreal.

In Fig 10(F), Zero-DCE recovers the indoor sports stadium image with distorted colors in the upper-right region, and the scene is dark. Fig 13(F) shows that the bookshelf part (row 3, column 2) appears a muddy shadow when Zero-DCE is used to recover the indoor image. The reason lies in that the Zero-DCE can only performs enhancement for the whole image, not for the specific areas in the image. In the strong reflection and extreme contrast conditions, the Zero-DCE work poorly in image enhancement, which is also shown in Fig 12(F).

Using the URetinex-Net, Fig 12(E) shows that the clouds in the image are almost not recovered. It is covered by over-enhanced white color. As shown in Fig 14(E), the details of the two chairs and the water bottle in the image are lost, the overall visual effect is blurry. In the URetinex-Net, the Retinex decomposition and reconstruction for the low-light image is directly performed. Therefore, the URetinex-Net cannot completely recover the extremely low-light images. There are still some problems such as distortion in recovering image details. These problems can affect image quality and visual effect.

From Figs 10–15, the network proposed in this paper performs better compared to the other five methods. It proves the effectiveness and generalization of the proposed network. They are more in line with the visual perception of the human eye.

### Objective metric evaluation

In the objective visual evaluation, several rigorous objective metrics are used to assess the performance comprehensively. These metrics include PSNR, SSIM, NIQE, PI, BRISQUE, and NIMA. Among these metrics, higher values for PSNR, NIMA, and SSIM indicate better image quality, while lower values for NIQE, PI, and BRISQUE indicate better visual image quality. The results with bold font in the table represent the best outcomes.

To maximize the accuracy, 15 images are selected on LOL dataset, 10 images are selected on the VE-LOL-L dataset, 10 images are selected on SID dataset, 10 images are selected on ELD dataset, 10 images are selected on DICM dataset, and 10 images are selected on MEF dataset as the test set. The average values of the six methods are calculated on different datasets. The experimental results are shown from Tables 2–7.

**Table 2:** Comparison results of different methods on LOL dataset.

Method	PSNR	SSIM	NIQE	PI	BRISQUE	NIMA
**KinD**	16.7743	0.7535	5.154	4.4734	28.8362	4.5491
**Retinex-Net**	16.774	0.425	8.8727	4.9546	51.8148	4.0981
**SCI**	14.784	0.5254	**4.8725**	4.5621	25.6625	4.5118
**URetinex-Net**	19.8414	0.7596	4.9838	**3.4375**	**15.9924**	4.5933
**Zero-DCE**	17.1061	0.558	7.9492	4.451	32.4807	4.3166
**ours**	**22.4568**	**0.8243**	5.1015	4.8759	19.2804	**4.7531**

**Table 3:** Comparison results of different methods on VE-LOL-L dataset.

Method	PSNR	SSIM	NIQE	PI	BRISQUE	NIMA
**KinD**	18.9565	0.8868	4.4806	3.3359	33.5243	4.413
**Retinex-Net**	17.3691	0.3567	9.847	5.4258	58.3901	4.0849
**SCI**	12.429	0.4095	**4.3896**	4.6529	32.2458	4.2986
**URetinex-Net**	19.9591	0.7188	5.9596	**3.1736**	34.7605	**4.6784**
**Zero-DCE**	17.4572	0.489	8.8368	4.8367	42.3082	4.2346
**ours**	**21.3067**	**0.8943**	5.1435	3.7925	**29.3789**	4.5057

**Table 4:** Comparison results of different methods on SID dataset.

Method	PSNR	SSIM	NIQE	PI	BRISQUE	NIMA
**KinD**	15.9137	0.4638	**4.0813**	3.0832	27.0418	3.6448
**Retinex-Net**	12.7897	0.1392	12.307	7.0149	56.6511	3.4739
**SCI**	14.4644	0.2332	10.2473	6.5421	50.4445	3.7729
**URetinex-Net**	**17.4981**	0.6548	9.56	5.5247	45.4748	3.875
**Zero-DCE**	14.4997	0.1974	11.1256	6.3478	52.7634	3.6217
**ours**	15.8412	**0.7664**	4.3545	**2.8832**	**26.0695**	**4.2032**

**Table 5:** Comparison results of different methods on ELD dataset.

Method	PSNR	SSIM	NIQE	PI	BRISQUE	NIMA
**KinD**	18.4923	0.7111	3.2804	3.2439	29.1691	4.7475
**Retinex-Net**	15.8025	0.5781	2.7454	**1.8824**	24.8244	4.3044
**SCI**	18.6216	0.6936	3.0955	2.6803	25.0041	**4.9917**
**URetinex-Net**	20.569	0.762	2.76	2.1829	20.2267	4.7735
**Zero-DCE**	20.0898	0.7568	3.0959	2.6429	38.2101	4.8092
**ours**	**22.7239**	**0.8525**	**2.6639**	3.0939	**18.2004**	4.4666

**Table 6:** Comparison results of different methods on DICM dataset.

Method	NIQE	PI	BRISQUE	NIMA
**KinD**	7.8135	5.0134	94.3479	4.8124
**Retinex-Net**	4.5126	2.8733	29.3987	4.6314
**SCI**	3.3576	2.8129	20.0405	4.8123
**URetinex-Net**	4.5002	3.2584	14.9468	**4.9471**
**Zero-DCE**	**2.5188**	2.6445	16.1858	4.75
**ours**	3.2154	**2.1752**	**14.2458**	4.6577

**Table 7:** Comparison results of different methods on MEF dataset.

Method	NIQE	PI	BRISQUE	NIMA
**KinD**	4.3972	3.5216	33.0152	4.8262
**Retinex-Net**	4.8911	3.1347	22.0831	4.5752
**SCI**	3.9972	2.8426	**14.7606**	4.8046
**URetinex-Net**	4.0918	3.1095	22.461	4.8796
**Zero-DCE**	**3.6696**	2.6292	19.2923	**4.9699**
**ours**	4.0276	**2.5957**	17.3762	4.758

The experimental results on the paired datasets LOL and VE-LOL-L are shown in Tables 2 and 3. As shown in Table 2, the proposed network achieves best values of 22.4568, 0.8243, and 4.7531 in PSNR, SSIM, and NIMA respectively. These values are 13.18%, 8.52%, and 3.5% higher compared with the second highest value. According to Table 3, the proposed network achieves best values of 21.3067, 0.8943, and 29.3789 in PSNR, SSIM, and BRISQUE respectively. The values of PSNR, SSIM and BRISQUE are 6.75%, 0.85% and 8.9% better compared with the second-best value.

The experimental results on the paired datasets SID and ELD are presented in Tables 4 and 5. Table 4 shows that in SSIM, PI, BQISQUE, and NIMA, our method improves 17.04%, 6.48%, 3.59%, and 8.47% compared to the second-best values. In Table 5 our method improves 10.48%, 11.88%, 2.97%, and 10.02% in PSNR, SSIM, NIQE, and BRISQUE compared to the second-best values.

The experimental results on the unpaired datasets DICM and MEF are shown in Tables 6 and 7. From Table 6, it can be seen that the proposed network achieves best values of 2.1752 and 14.2458 in PI and BRISQUE, respectively. These values are 17.74% and 4.68% better than the second-best value. As shown in Table 7, the proposed network achieves best value of 2.5957 in PI. Although the values of metrics except PI are not optimal, the result achieves a more balanced performance.

### Ablation study

**Fig 16:**
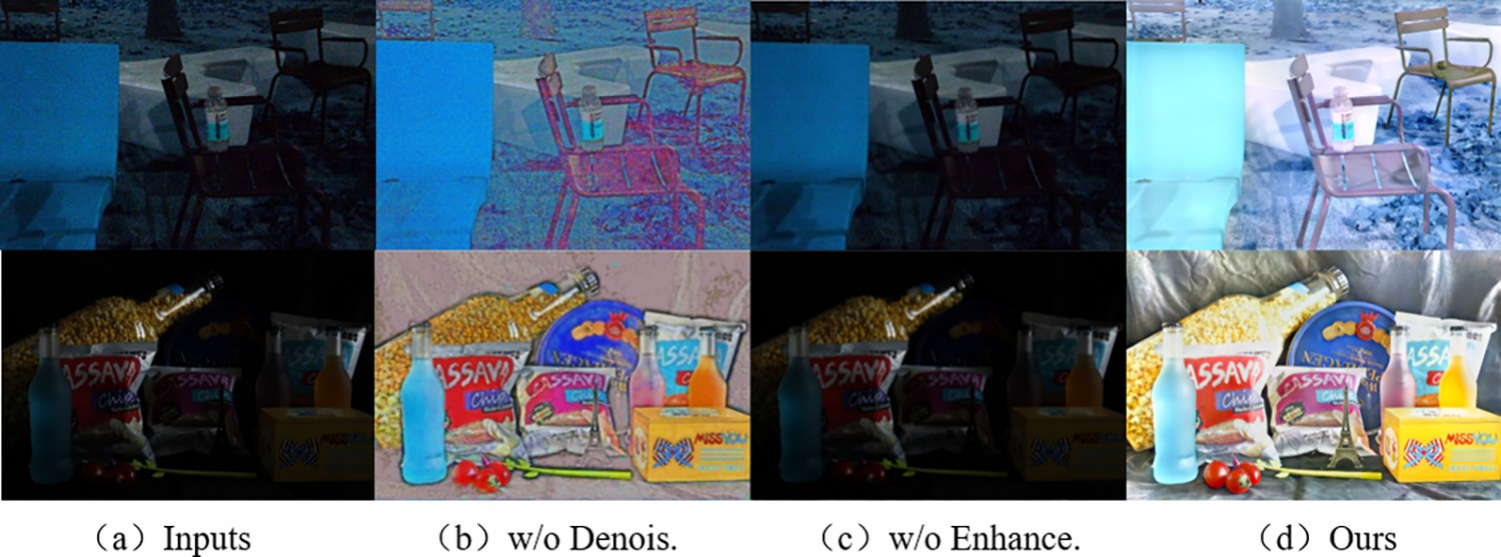
Ablation studies on the framework. ‘w/o Denois.’ denotes our method without reflective component denoising network. ‘w/o Enhance.’ denotes our method without illumination component enhancement network.

To verify the necessity of the method framework proposed in this paper, we conducted ablation experiments by separately removing the denoising network and the enhancement network. The results of the experiments are shown in Fig 16 and Table 8.

**Table 8:** Comparison of ablation results for different modules.

Enhancement network	Denoising network	PSNR	SSIM	NIQE	PI	BRISQUE	NIMA
**✓**	**Ο**	15.8937	0.4655	6.7658	5.9814	50.4253	3.5079
**Ο**	**✓**	9.94	0.3343	6.8481	6.014	55.5266	3.564
**✓**	**✓**	**19.4325**	**0.7643**	**4.425**	**2.1453**	**23.0573**	**4.5649**

From Fig 16(B), it can be observed that when the enhancement sub-network is present but the denoising sub-network is absent, the overall image exhibits excessive noise and unclear details, although there is some improvement in brightness. From Fig 16(C), it can be seen that when the denoising sub-network is present but the enhancement sub-network is absent, the image noise is effectively reduced, but the overall brightness is hardly enhanced, resulting in a dull color appearance. In Fig 16(D), the contrast of the image is effectively improved, with clearer details and noticeable noise reduction. This clearly has shown that all the components are important for achieving better performance.

The objective comparisons of ablation results for each module are presented in Table 8. It can be seen that both the absence of the denoising network or the enhancement network leads to relatively poor performance across multiple metrics. In contrast, our proposed network achieves the best results across all metrics, further demonstrating the effectiveness of the method proposed in this paper.

## Conclusions

In order to further improve the effect of low-light image enhancement, a Retinex-based image enhancement network for a low-light environment is proposed. A new loss function, CA attention mechanism and the adaptive dynamic iteration method is introduced in the proposed network. Experiments show that most objective metrics have been improved. At the same time, the proposed network has a better denoising effect and the visual effect is more in line with human eye vision. It proves the effectiveness and generalization of the network proposed in this paper.

## Supporting information

S1 File (ZIP)
