# Supplementary figures and images for: A Retinex-based network for image enhancement in low-light environments
Source: PLoS One. 2024 May 24;19(5):e0303696. doi: 10.1371/journal.pone.0303696 (PMC11125518; doi:10.1371/journal.pone.0303696)

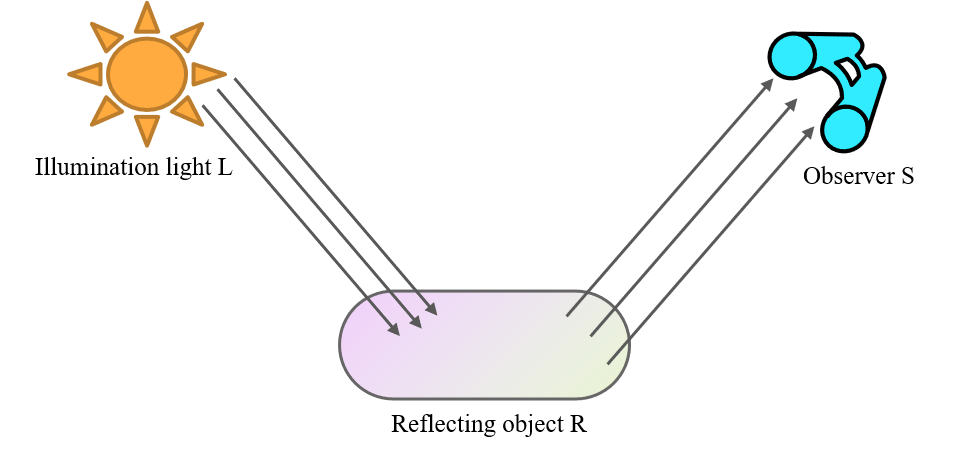

Supplement: S1 File — (ZIP) [file pone.0303696.s001.zip › Supporting Information/Fig1.tif]

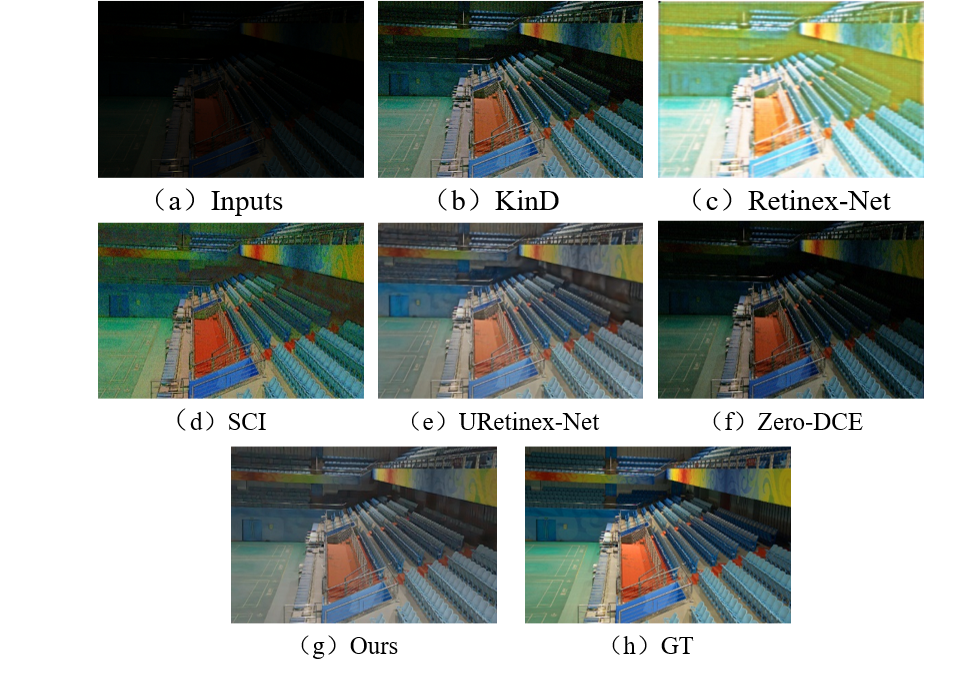

Supplement: S1 File — (ZIP) [file pone.0303696.s001.zip › Supporting Information/Fig10.tif]

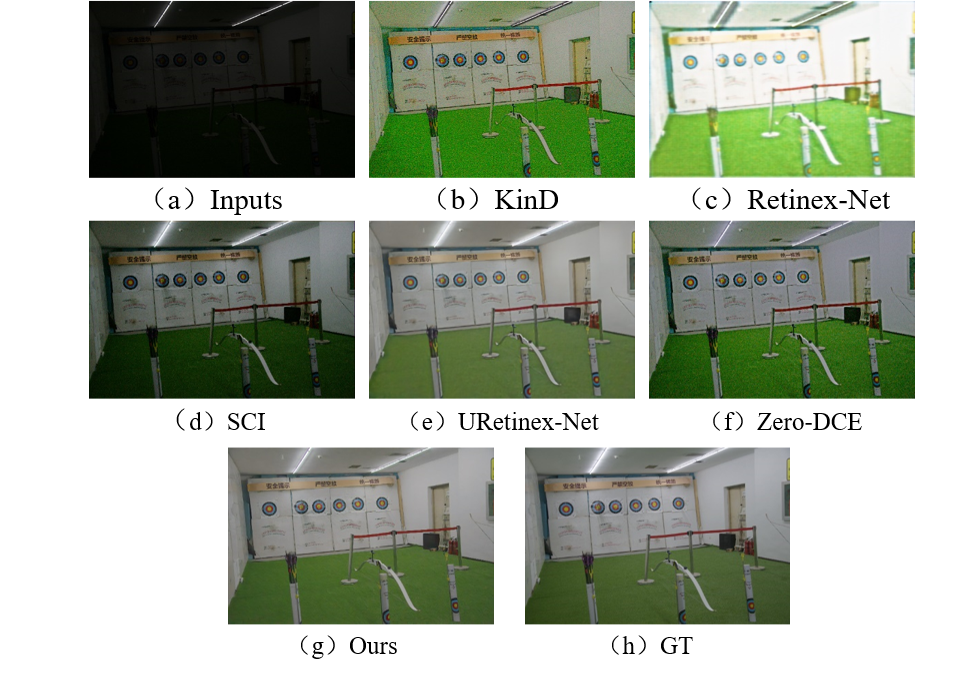

Supplement: S1 File — (ZIP) [file pone.0303696.s001.zip › Supporting Information/Fig11.tif]

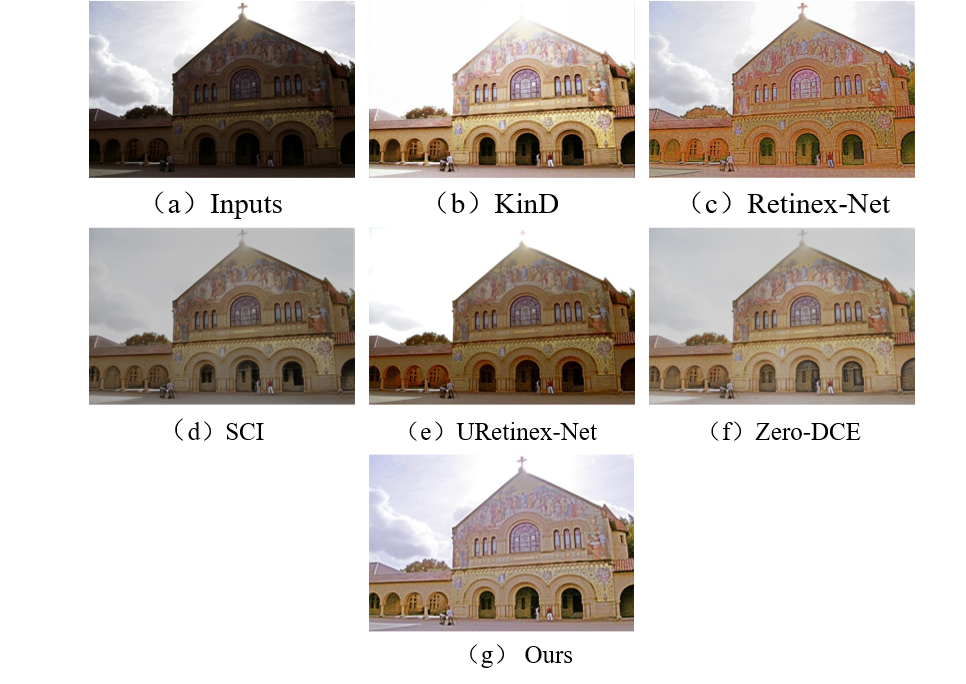

Supplement: S1 File — (ZIP) [file pone.0303696.s001.zip › Supporting Information/Fig12.tif]

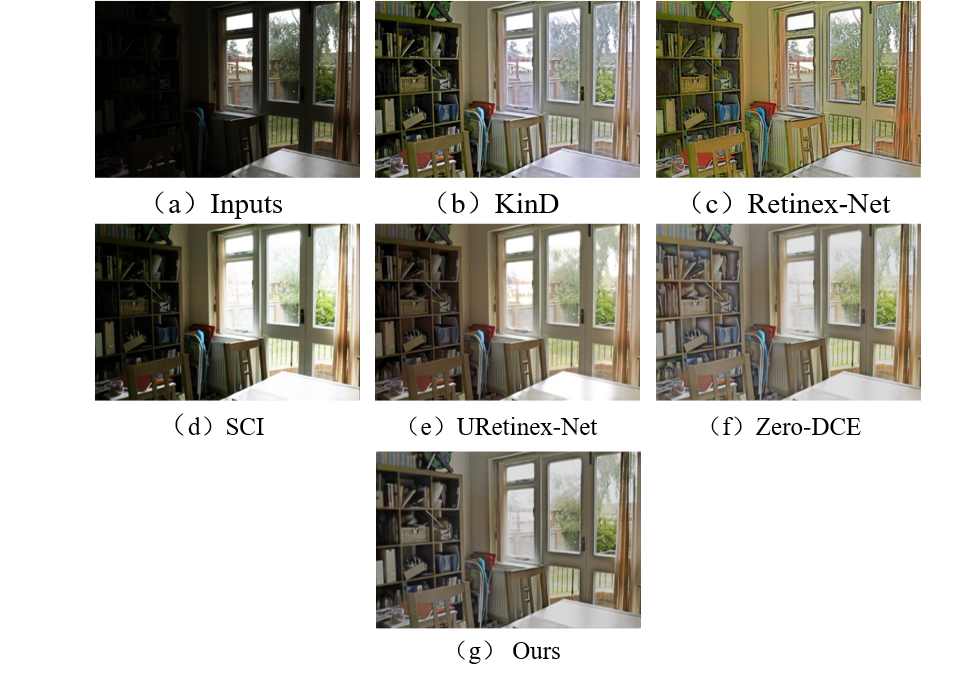

Supplement: S1 File — (ZIP) [file pone.0303696.s001.zip › Supporting Information/Fig13.tif]

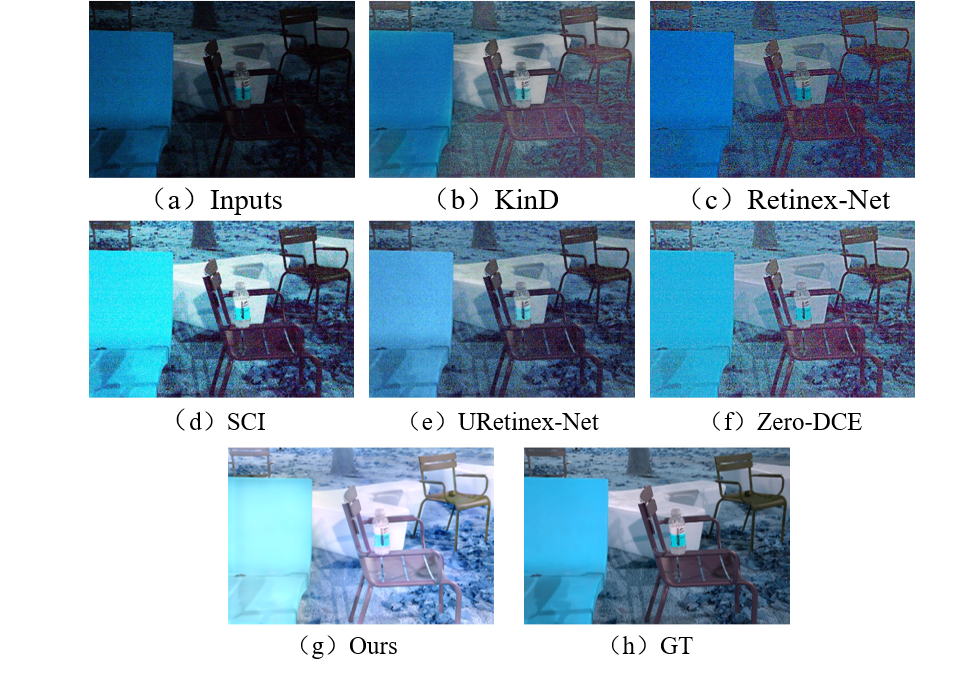

Supplement: S1 File — (ZIP) [file pone.0303696.s001.zip › Supporting Information/Fig14.tif]

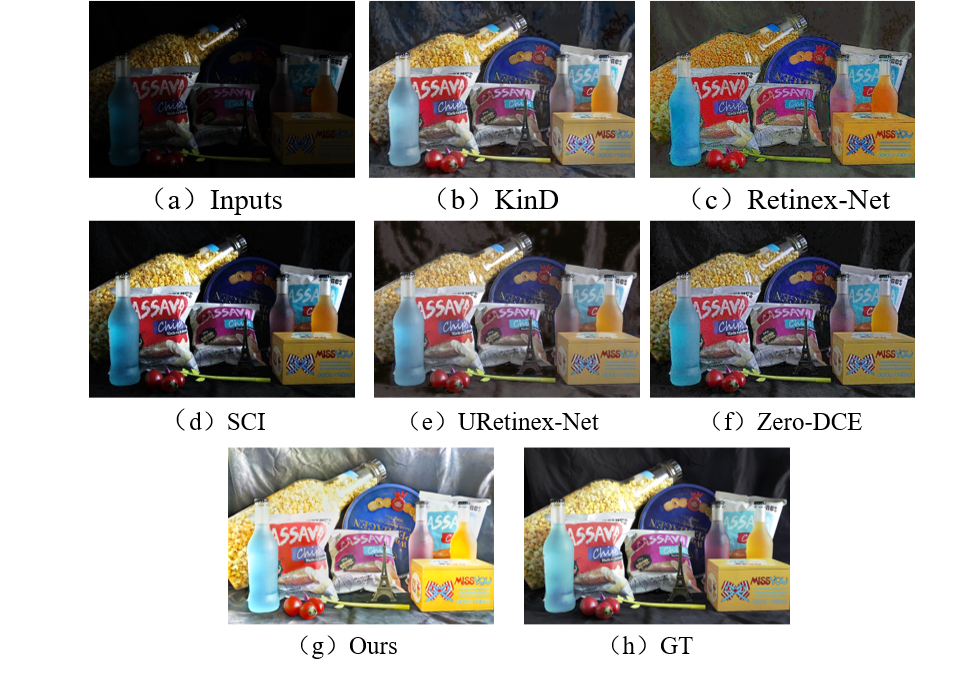

Supplement: S1 File — (ZIP) [file pone.0303696.s001.zip › Supporting Information/Fig15.tif]

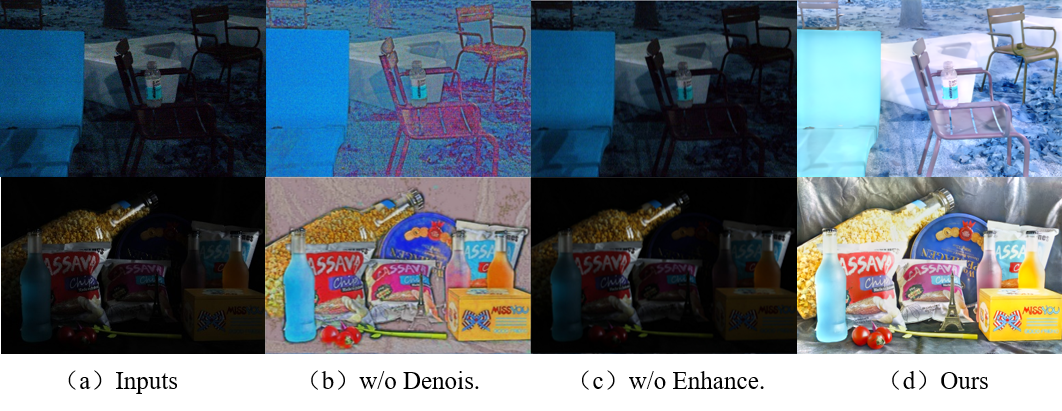

Supplement: S1 File — (ZIP) [file pone.0303696.s001.zip › Supporting Information/Fig16.tif]

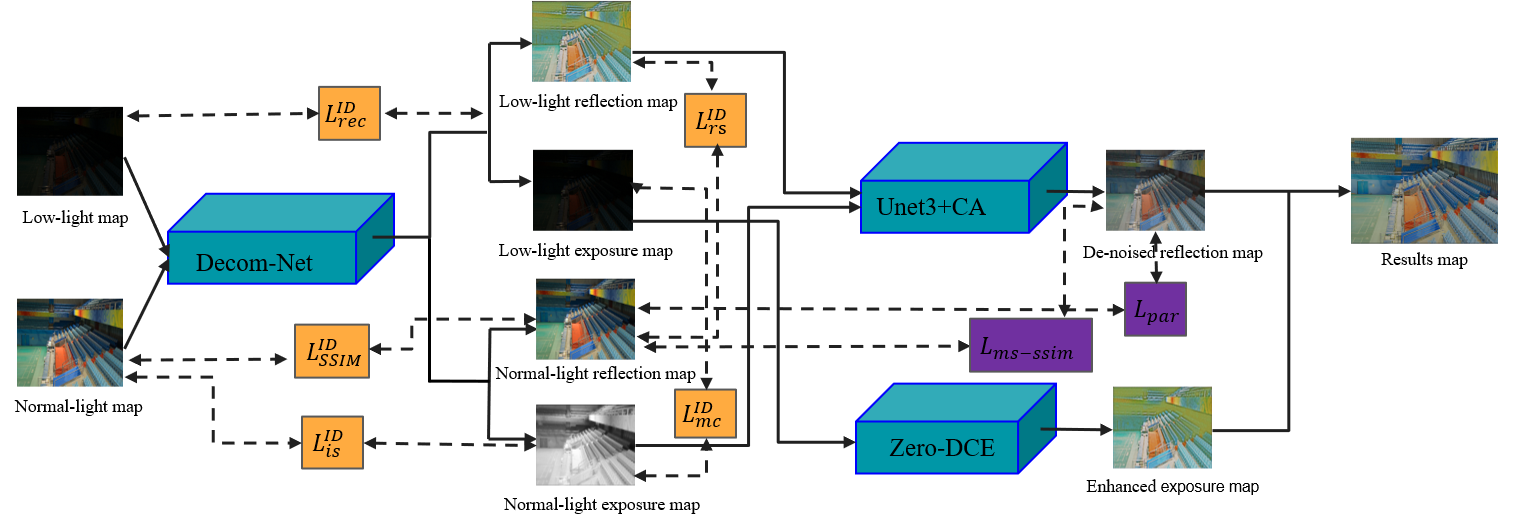

Supplement: S1 File — (ZIP) [file pone.0303696.s001.zip › Supporting Information/Fig2.tif]

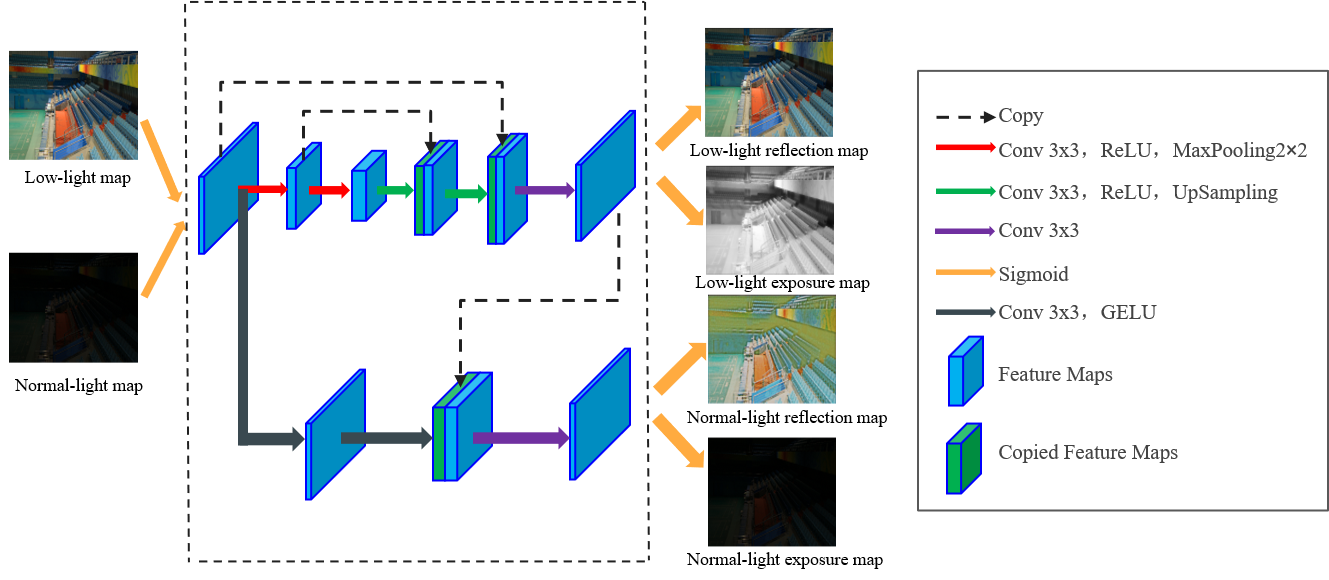

Supplement: S1 File — (ZIP) [file pone.0303696.s001.zip › Supporting Information/Fig3.tif]

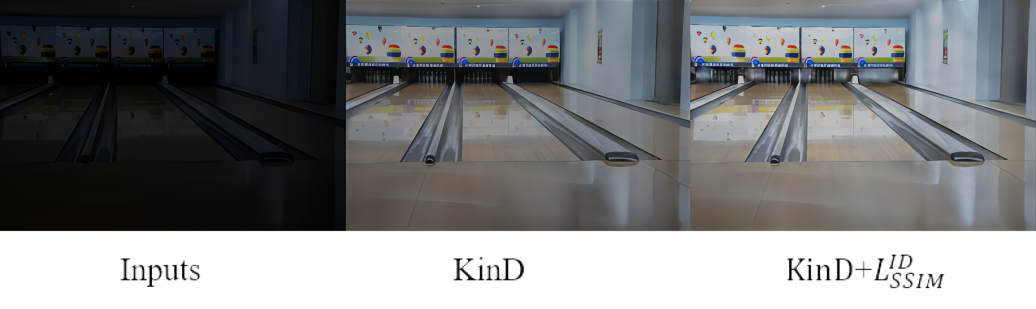

Supplement: S1 File — (ZIP) [file pone.0303696.s001.zip › Supporting Information/Fig4.tif]

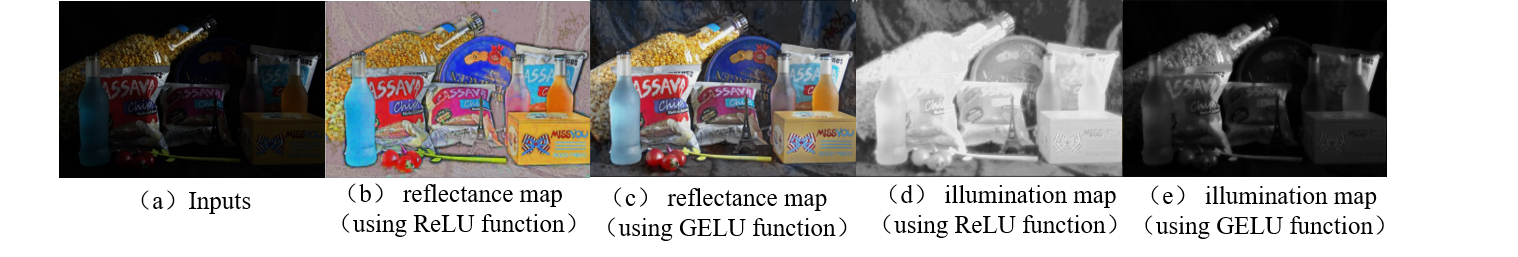

Supplement: S1 File — (ZIP) [file pone.0303696.s001.zip › Supporting Information/Fig5.tif]

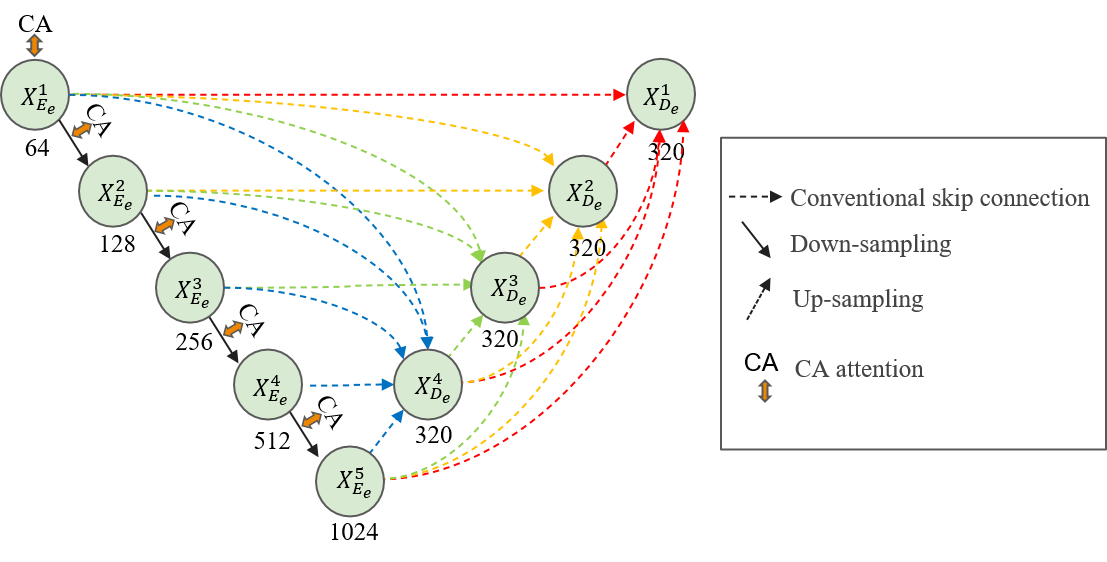

Supplement: S1 File — (ZIP) [file pone.0303696.s001.zip › Supporting Information/Fig6.tif]

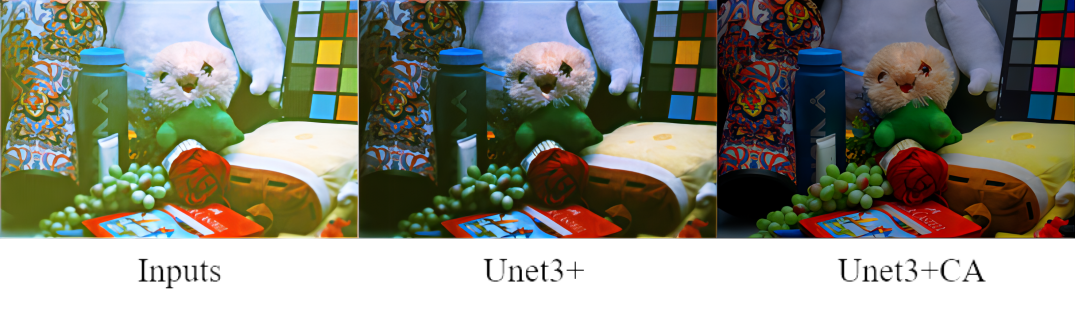

Supplement: S1 File — (ZIP) [file pone.0303696.s001.zip › Supporting Information/Fig7.tif]

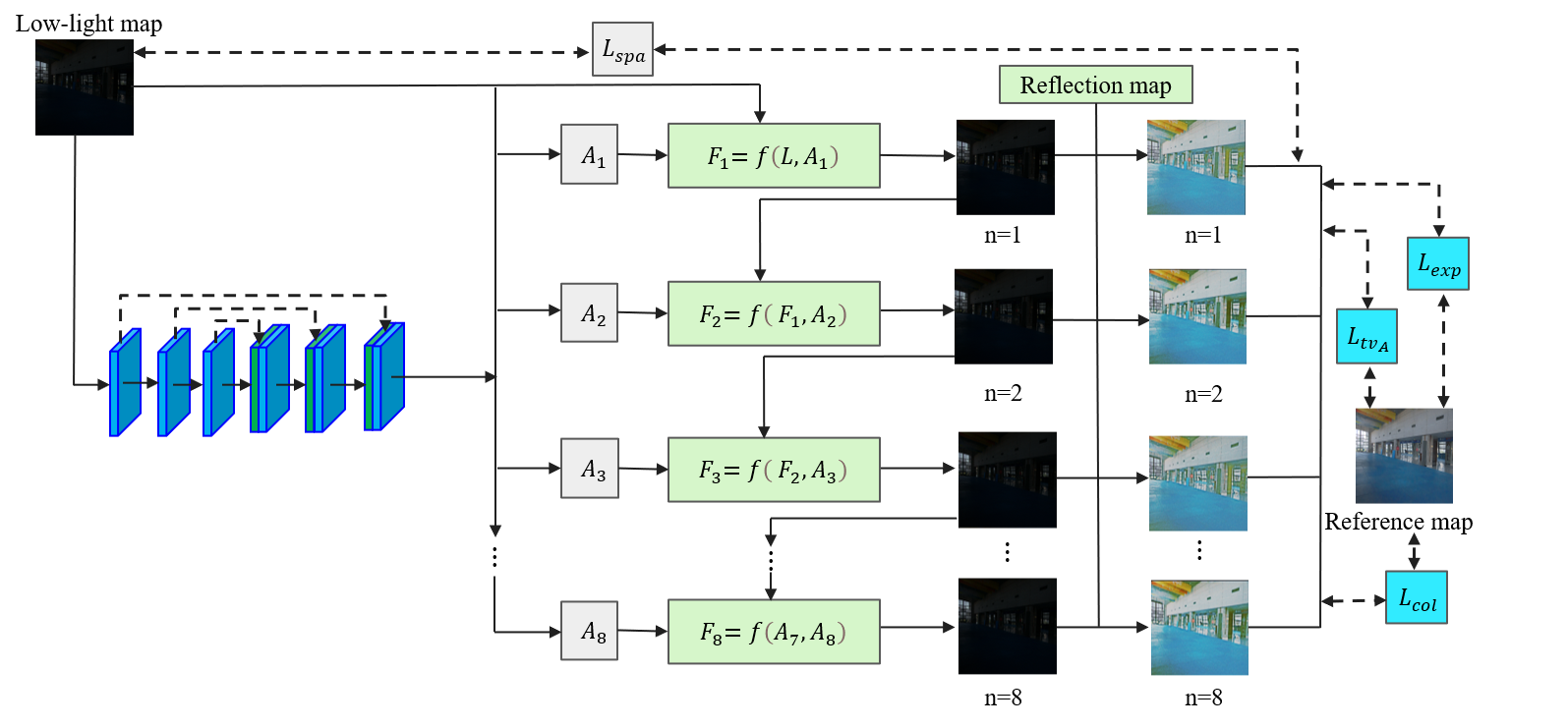

Supplement: S1 File — (ZIP) [file pone.0303696.s001.zip › Supporting Information/Fig8.tif]

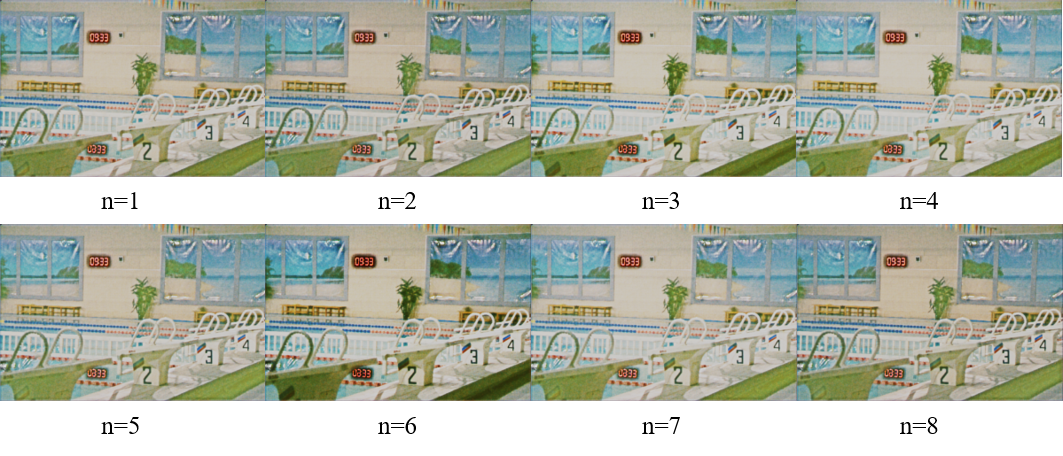

Supplement: S1 File — (ZIP) [file pone.0303696.s001.zip › Supporting Information/Fig9.tif]
